# Why Multitarget Vasodilatory (Endo)cannabinoids Are Not Effective as Antihypertensive Compounds after Chronic Administration: Comparison of Their Effects on Systemic and Pulmonary Hypertension

**DOI:** 10.3390/ph15091119

**Published:** 2022-09-07

**Authors:** Patryk Remiszewski, Barbara Malinowska

**Affiliations:** Department of Experimental Physiology and Pathophysiology, Medical University of Bialystok, Mickiewicza Str. 2A, 15-222 Białystok, Poland

**Keywords:** (endo)cannabinoids, systemic hypertension, pulmonary hypertension

## Abstract

Systemic and pulmonary hypertension are multifactorial, high-pressure diseases. The first one is a civilizational condition, and the second one is characterized by a very high mortality rate. Searching for new therapeutic strategies is still an important task. (Endo)cannabinoids, known for their strong vasodilatory properties, have been proposed as possible drugs for different types of hypertension. Unfortunately, our review, in which we summarized all publications found in the PubMed database regarding chronic administration of (endo)cannabinoids in experimental models of systemic and pulmonary hypertension, does not confirm any encouraging suggestions, being based mainly on in vitro and acute in vivo experiments. We considered vasodilator or blood pressure (BP) responses and cardioprotective, anti-oxidative, and the anti-inflammatory effects of particular compounds and their influence on the endocannabinoid system. We found that multitarget (endo)cannabinoids failed to modify higher BP in systemic hypertension since they induced responses leading to decreased and increased BP. In contrast, multitarget cannabidiol and monotarget ligands effectively treated pulmonary and systemic hypertension, respectively. To summarize, based on the available literature, only (endo)cannabinoids with a defined site of action are recommended as potential antihypertensive compounds in systemic hypertension, whereas both mono- and multitarget compounds may be effective in pulmonary hypertension.

## 1. Introduction

Systemic and pulmonary hypertension are multi-factorial, high-pressure diseases that influence the left and right parts of the heart, respectively. The first one is a civilizational condition that affects one out of every three adults worldwide. The second one impacts only a fraction per thousand of the population but has a very high mortality rate. Treatment resistance and low effectiveness make searching for new therapeutic strategies an important task. Among many others, (endo)cannabinoids are proposed as a possible drug for different types of hypertension. In this review, we inspected this thesis.

## 2. Systemic Hypertension

Systemic arterial hypertension, commonly known as hypertension, is a multifunctional disease characterized by persistently increased blood pressure (BP) in the systemic arteries, with values over 140 mmHg for systolic BP (SBP) and over 90 mmHg for diastolic BP (DBP) [1,2,3]. Most cases of hypertension (90–95%) are classified as primary or essential hypertension with a multifactorial genetic–environmental etiology. The remaining cases are those with identified causes (e.g., renal artery stenosis, pheochromocytoma, adrenal adenoma, or single-gene mutations), known as secondary hypertension [1,3]. Among the main risk factors connected to primary hypertension, many (high sodium and low potassium intake, alcohol consumption, lack of physical activity, overweight and obesity, unhealthy diet, and smoking) can be altered by patients [4].

Estimates show that more than 1.3 billion people (around 30% of adults) suffer from hypertension worldwide. In some countries where the threshold of hypertension has been lowered to ≥130/80 mmHg (e.g., the USA and China), the prevalence increased to about 45% of the adult population [4]. Hence, it should be no surprise that this disease is considered the most critical and expensive public health problem and is the leading single modifiable contributor to all-cause mortality and disability worldwide, responsible for more than 9 million deaths annually [1,3]. Even a small decrease in elevated BP can significantly reduce the risk of major adverse cardiovascular events and death [2].

The pathophysiological basis of hypertension is complex and consists of the interplay between renal, humoral, vascular, and central mechanisms that normally maintain physiological BP, but their malfunction or disruption eventually leads to elevated cardiac output, body fluid volume, and/or peripheral resistance [1,5]. Aside from the predominant significance of enhanced sympathetic tone in the development and progression of hypertension [6], one of the most crucial components of its pathogenesis is the renal renin–angiotensin–aldosterone system (RAAS), which regulates BP by mediating sodium retention, natriuresis, and vasoconstriction [7]. In addition, the vasculature of patients with hypertension is less responsive to vasodilatation and may be remodeled, stiffened, and affected by inflammatory and oxidative changes [8].

The basic first-line treatment of hypertension is based on three main pathways and includes (1) angiotensin-converting enzyme inhibitors, (2) angiotensin receptor antagonists, (3) calcium channel blockers, and (4) diuretics. It is recommended that therapy for hypertension should be carried out, even started, as combined therapy with two or more substances acting by different mechanisms. To provide individualized therapy, other groups are often added to the primary groups, such as β-blockers, mineralocorticoid antagonists, α-blockers, α_2_-agonists, direct vasodilators, or renin inhibitors [2]. Despite the wide selection of antihypertensive drugs, there are still around 10–20% cases of treatment-resistant hypertension associated with a higher impact on cardiovascular risk [9] and cases where proper treatment cannot be administered due to the unacceptable side effects of currently available therapies. Drugs directed at novel mechanisms are therefore being sought [1].

## 3. Pulmonary Hypertension

Pulmonary hypertension (PH) is a rare progressive cardiopulmonary disease characterized by increased pulmonary arterial pressure, which leads to right heart failure and, consequently, premature death. For many years, PH has been defined as mean pulmonary arterial pressure (mPAP) ≥ 25 mmHg. Population studies have shown that the average mPAP in healthy individuals is about 14 mmHg and rarely exceeds 19 mmHg [10]. Elevated pulmonary pressure, up to 19–25 mmHg, increases mortality and the further risk of developing full-blown PH [11,12,13,14,15]. The search for a borderline between “normal” and elevated pressure in pulmonary circulation led, in 2018 [14], to a proposal for a new frontier of the PH of mPAP ≥ 20 mmHg (i.e., two standard deviations above mean pressure) obtained with right heart catheterization. Further hemodynamic classification into pre-capillary PH, isolated post-capillary PH, or combined pre- and post-capillary PH is carried out using values of pulmonary vascular resistance (PVR) and pulmonary arterial wedge pressure (PAWP) [16].

Classification of PH is based on similar histology and pathophysiology but also concurrent treatment strategies and responses to them [13]. The World Health Organization lists five clinical groups: (1) pulmonary arterial hypertension (PAH); (2) PH related to left-sided heart disease; (3) chronic lung disease-related PH; (4) chronic thromboembolic PH; (5) other types of PH [17]. Groups 2 and 3 are the most common (millions of patients worldwide); however, the greatest emphasis is placed on the rarest types, i.e., groups 1 and 4 [16,18]. The epidemiology of PAH is not easy to determine precisely, but currently available data allow us to estimate its incidence at around 5.8 and prevalence at around 51 cases per million [19]. It should be kept in mind that these statistics were made according to the 2003 PH/PAH definition, and the values will probably increase by up to 10% after the mPAP threshold is lowered [20]. The greatest problem with PAH, however, is still high mortality. With the absence of treatment, the average survival of patients in the 1990s was 2.8 years, whereas, with pharmacological intervention, it is now about 7 years [21]. Survival rates are also connected to patient risk profiles. At baseline, the 1-year, 3-year, and 5-year survival rates are approximately 98, 90, and 80% in the low-risk group, 87, 68, and 52% in the intermediate-risk group, and 75, 52, and 33% in the high-risk group, respectively [22,23]. Even though PAH may be caused by well-known factors, such as toxins and drugs (e.g., methamphetamine), HIV infection, schistosomiasis, connective tissue disease, or congenital heart disease, most cases (up to 67%) are of unknown origin (idiopathic) [24].

The pathophysiology of PAH is complex and primarily connected to the vascular remodeling of the three layers of the small distal pulmonary arteries, which results in their obliteration, muscularization, and the formation of characteristic plexiform lesions. All of those changes led to progressive narrowing of blood vessels and increased mPAP and PVR (all cases of PAH are hemodynamically classified as pre-capillary with PVR ≥ 3 Wood units) [15,18,24]. Vascular and perivascular inflammation and fibrosis play important roles in the process [25]. As the vessel’s changes progress, the right part of the heart must take on an increasing burden. The right ventricle (RV) undergoes hypertrophy, dilatation, fibrosis, inflammation, ischemia, and metabolic disturbances. In the initial phase, RV remodeling remains adaptive with preserved hemodynamic function; however, at some point, it can no longer keep up with the vasculopathy and transforms into a maladaptive phenotype [18,26].

Currently, specific treatment is available mostly for PH groups 1 and 4. In PAH, three main regulatory pathways are the targets of therapy focused on vasodilatation of pulmonary arteries only: (1) nitric oxide (NO)-cyclic guanosine monophosphate (cGMP) pathway with phosphodiesterase type 5 (PDE5) inhibitors (sildenafil, tadalafil) and a soluble guanyl cyclase (sGC) stimulator (riociguat); (2) prostacyclin (PGI_2_)-cyclic adenosine monophosphate (cAMP) pathway with PGI_2_ analogs (epoprostenol, treprostinil, iloprost) and receptor agonist (selexipag); and (3) endothelin receptor pathway with its antagonists (bosentan, macitentan, ambrisentan) [27]. Most patients with PAH receive more than one drug as up-front combination therapy, which is now the standard of care [15,16,18]. However, none of the currently available therapeutic options can cure PAH, and life expectancy, despite significantly increasing in recent years, is unsatisfactory. Moreover, PAH is a multifactorial disease, and pulmonary vasoconstriction as the primary target of current therapies seems deficient. Therefore, the search for new potential drug targets is extremely important in the case of PAH.

## 4. Animal Models of Hypertension

Clinical trials and meta-analyses are the most valuable sources of knowledge about the most efficient treatment strategies for every kind of hypertension. However, animal models are needed for preclinical studies to discover the specific genetic, cellular, and molecular mechanisms underlying the disease or to test novel therapeutic strategies. As the human pathophysiology of hypertension differs among individuals, it is difficult to create a model that ideally mimics all disturbances [28,29,30].

Among animal models of systemic hypertension, there are two main groups. Models based on genetic alterations (both mono- and polygenic), which are closest to essential human hypertension, and those in which hypertension is induced by the researcher’s interventions (dietary, pharmacological, and/or surgical), corresponding to secondary hypertension. The most important models of hypertension covered in this review are presented in Table 1. The most frequent model is spontaneously hypertensive rats (SHR); another polygenic model is Dahl salt-sensitive rats. Induced models are most often represented in publications by three types: angiotensin II (Ang-II), L-N^G^-nitro arginine methyl ester (L-NAME, the inhibitor of nitric oxide synthase (NOS)), and deoxycorticosterone acetate (DOCA)-salt models [28,29]. In addition to the most widely used models, many others that reflect some features of hypertension can be found, such as TGR(mRen2)27, in which overexpression of the renin gene is induced, adrenal regeneration hypertension (ARH), in which contralateral adrenal enucleation is performed, and metacorticoid hypertension, which is similar to DOCA-salt but with more stable development of hypertension or renal hypertension (so-called two-kidney, one clip (2K1C), where the renal artery is constricted [29,31,32]. New methods are continuously being developed. For example, recently, two new models of rapid induction of multifactorial heart disease associated with hypertension (SHR and 2K1C), hypothyroidism, and a high-fat diet were introduced [30].

Similar to systemic hypertension, no single animal PH model is likely to be universally appropriate. The classical models are the ones in which PH is induced by the administration of alkaloid, monocrotaline (MCT), or chronic hypoxia. However, the direct toxic effects of MCT on various organs, including the liver and heart, represent a serious limitation of the MCT model [33,34,35]. Exposure to chronic hypoxic conditions leads to the induction of PH, similar to many PH-causing conditions in humans (e.g., chronic obstructive pulmonary disease). Additional administration of vascular endothelial growth factor (VEGF) receptor antagonist (Sugen) results in severe and irreversible changes (in rats, but not mice) [28,34,36]. In addition to the classic models of PH, more attention is paid to models with a genetic basis, including monogenic ones [37].

## 5. Cannabinoids as a Potential New Therapy against Systemic and Pulmonary Hypertension

As mentioned in the previous sections, there is still a need for new effective pharmacotherapy against both systemic and pulmonary hypertension. In recent years, scientists, physicians, and patients have paid increasing attention to (endo)cannabinoids, including medical marijuana, since the therapeutic potential of the endocannabinoid system is enormous and is based on all groups of cannabinoids. Thousands of scientific papers, hundreds of clinical trials, and a few approved drugs (Sativex, Marinol, Syndros, Cesamet, and Epidiolex) provide proof of this potential [38,39,40,41,42,43,44]. Moreover, one of the potential targets of cannabinoid-based therapy is the cardiovascular system, including systemic and pulmonary hypertension, as was stated in reviews over the last few years [45,46,47,48,49,50,51,52,53,54,55,56,57]. Such promising conclusions are based mainly on three aspects: (1) the strong vasodilatory effects of (endo)cannabinoids [58,59]; (2) the overactivation of endocannabinoid tone in hypertension [38,46], and (3) stronger hypotensive responses in hypertensive animals than in normotensive controls [46]. However, results regarding the beneficial effects of (endo)cannabinoids are based on in vitro experiments or in vivo ones after acute intravenous (*i.v.*) injection of compounds in anesthetized animals. Thus, the present review was aimed at determining (based on the available literature) the effects of chronic administration of (endo)cannabinoids on BP in various models of systemic and pulmonary hypertension. Moreover, we compared changes in the heart, arteries, kidneys, brain, blood, and lungs (if applicable) (i.e., organs/tissues important for the development of the above types of hypertension) and the liver to determine whether particular changes are tissue-dependent. We focused on changes in functional cardiac and vessel (mainly endothelial-dependent) responses, components of the endocannabinoid system, and markers of oxidative stress and inflammation since, according to the modified Dr. Page’s Mosaic Theory of hypertension [8], hypertension is the result of many factors, including, among others, cardiac output [60], vascular reactivity (mainly endothelial-dependent) [61], oxidative stress [62], and inflammation [63], which interact to raise BP and cause end-organ damage.

## 6. Cannabinoids and the Endocannabinoid System

**Figure 1:**
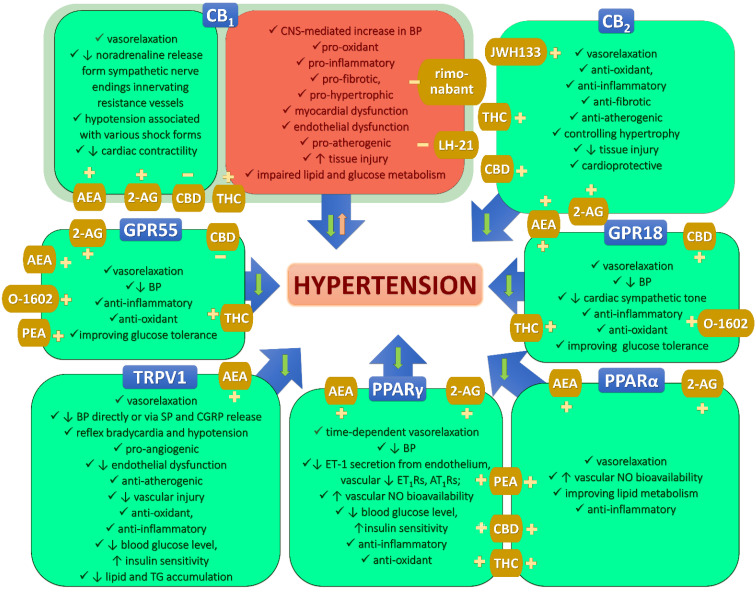
Well-known potential effects (not only related to the cardiovascular system) of compounds described in tables after interacting with classical and non-classical cannabinoid receptors. For receptor affinity, see [74,75,76,77,78]. For references regarding effects of particular receptors, see Section 6. Green indicates pro-hypotensive and red pro-hypertensive effects. Arrows next to effects indicate increase (↑) or decrease (↓); arrows in the center indicate predominantly pro-hypotensive (↓) or hypertensive (↑) effects. (+) activation, (−) blockade. 2-AG—2-arachidonoyl glycerol; AEA—anandamide; AT_1_Rs—angiotensin II type 1 receptors; BP—blood pressure; CB_1_—cannabinoid type 1 receptor; CB_2_—cannabinoid type 2 receptor; CBD—cannabidiol; CGRP—calcitonin gene-related peptide; CNS—central nervous system; ET-1—endothelin 1; ET_1_Rs—endothelin 1 receptors; NO—nitric oxide; PEA—palmitoyl ethanolamide; PPAR—peroxisome proliferator-activated receptor; SP—substance P; THC—Δ^9^-tetrahydrocannabinol; TG—triglycerides; TRPV1—transient receptor potential vanilloid 1.

Cannabinoids are a group of compounds that were first isolated from *Cannabis sativa*. The most abundant plant-derived molecules from this group are Δ^9^-tetrahydrocannabinol (THC), Δ^8^-tetrahydrocannabinol (Δ^8^-THC), cannabidiol (CBD), cannabinol (CBN), cannabigerol (CBG), cannabichromene (CBC), Δ^9^-tetrahydrocannabivarin (THCV), cannabivarin (CBV), and cannabidivarin (CBDV) [64]. It was not until the early 2000s that there was increased interest in other phytocannabinoids, including non-intoxicating CBD. Forty years of research into the mechanism of action of THC led to the discovery of cannabinoid receptors (CBRs), along with their endogenous ligands and metabolic enzymes, which together form the endocannabinoid system. Currently, we distinguish three main groups of cannabinoids: (1) the phytocannabinoids listed above; (2) synthetic cannabinoids, including WIN55212-2, CP55940, and JWH133; and (3) endocannabinoids (eCBs), which are produced endogenously and have an affinity to classical CBRs or endocannabinoid-like compounds. Despite their similar chemical structure to eCBs, the latter compounds hardly bind to classical CBRs but can interact with other elements of the endocannabinoid system. The best-known eCBs are anandamide (AEA) and 2-arachidonoylglycerol (2-AG), whereas noladin ether (2-AGE), 2-linoleoylglycerol (2-LG), N-arachidonoyl-L-serine (ARA-S), dihomo-γ-linolenoyl ethanolamide (DGLEA), docosahexaenoyl ethanolamide (DHEA), eicosapentaenoyl ethanolamide (EPEA), homo-γ-linolenyl ethanolamide (HEA), linolenoyl ethanolamide (LEA), N-arachidonoyl dopamine (NADA), N-arachidonoyl glycine (NAGly), oleamide, oleoyl ethanolamide (OEA), palmitoyl ethanolamide (PEA), palmitoleoyl ethanolamide (POEA), stearoyl ethanolamide (SEA), and virodhamine are endocannabinoid-like compounds [38]. Among them, PEA and OEA are gaining popularity in the scientific community because of their beneficial effects, such as anti-inflammatory, anti-anaphylactic, analgesic, and hypophagic activity, as well as maintenance of glucose homeostasis [65]. Moreover, for decades, PEA, a plant-derived dietary supplement or nutraceutical, has been considered to have immunomodulatory properties [66,67,68].

(Endo)cannabinoids act via two types of G protein-coupled receptors (GPCRs), cannabinoid receptor CB_1_ (CB_1_R) and CB_2_ (CB_2_R). CB_1_Rs are spread all over the body but are mostly found in the central nervous system (CNS), which is the reason for the psychoactivity of THC. As shown in Figure 1, their activation exerts both pro-hypotensive and pro-hypertensive activity [39,45,46,69,70,71]. The hypotensive effects result mainly from a decrease in noradrenaline release from the sympathetic nerve endings innervating resistance vessels by the activation of presynaptic CB_1_Rs and direct vasodilatory effects determined in various (but not all) vessels [46,58]. However, it should be remembered that CB_1_Rs are also known for their pro-oxidant and pro-inflammatory effects, and their activation in the CNS leads mainly to a pressor response [39,45,46,71,72]. The highest density of CB_2_Rs occurs in the immune system. In contrast to CB_1_Rs, stimulation of CB_2_Rs leads to anti-inflammatory and anti-oxidant influences and other antihypertensive effects [73].

Apart from the classical ones, many different receptors may interact with both endo- and exogenous cannabinoids, such as orphan receptors GPR18 and GPR55, ionotropic transient receptor potential vanilloid type 1 (TRPV1), and peroxisome proliferator-activated receptors (PPARs) [79,80]. AEA is an endogenous ligand of TRPV1 receptors, the activation of which causes vasodilatation and other actions, leading to a decrease in BP (Figure 1) [69,81,82,83]. As shown in Figure 1, activation of GPR18 [84,85,86], GPR55 [77,87,88], PPARγ [75,89,90,91,92], or PPARα [89,91,92] can also lead to a drop in BP. Importantly, all of the above receptors are also present in the vascular and cardiac systems.

Despite slight variations by strain and vessel type, most cannabinoid receptors are expressed in both endothelium and smooth muscle cells of systemic vessels; however, sometimes, their expression/staining is more pronounced in endothelial cells [93,94,95]. The expression of GPR18 receptors in peripheral blood vessels is still a subject of debate [86]. CB_1_Rs, CB_2_Rs, TRPV1, GPR18, and GPR55 receptors are also expressed in pulmonary arteries (mostly evidenced in human studies), predominantly in the whole vessel wall, although some papers show an increased presence of CB_1_Rs in smooth muscle cells or, inversely, a prevalence of GPR18 receptors in the endothelium and adventitial layer of the vessel [48,93]. There are practically no studies comparing expression levels between systemic and pulmonary circulation, and most studies show a similar distribution of cannabinoid receptors throughout the vessels in both.

Cardiac CBRs are also widely distributed. CB_1_Rs and CB_2_Rs are present in the left ventricle, left and right atrium, and epicardial adipose tissue in humans and animals. GPR55 and GPR18 receptors were found in the left ventricle. Except for cardiac muscle tissue, CBRs are also present in coronary arteries but are absent from the electrical conduction system of the heart [78].

Due to the short biological half-life of eCBs, much attention is paid to their degradation process. Two main enzymes responsible for the catalysis of CBR ligands are fatty acid amide hydrolase (FAAH) (AEA and partially 2-AG) and monoacylglycerol lipase (MAGL) (mostly 2-AG). Their respective inhibitors, URB597 and JZL195, are used to enhance the endocannabinoid tone [38,80].

## 7. Vasodilatory Effects of Chosen (Endo)cannabinoids

As mentioned above, the strong vasodilatory effect of (endo)cannabinoids is one of the reasons they are suggested to possess potential anti-hypertensive and cardio- or vasculoprotective activity [58,59,69]. Table 2 presents the vasodilatory effects of all compounds examined in chronic experiments on hypertensive models (for descriptions, see Section 9, Section 10 and Section 11), which were examined in both normo- and hypertensive conditions in vitro. Indeed, as shown in Table 2, AEA (as well as its stable analog, methanandamide (MethAEA)), CBD, and THC exert direct vasodilatory effects. Importantly, their vasorelaxant action shows higher efficacy (up to 100% maximal effect) in resistance (mesenteric bed and small mesenteric arteries (sMAs)) [93,96,97,98,99], but much lower (up to 20%) in conductive systemic vessels (aorta, superior mesenteric arteries) [96,99,100]. One paper reported stronger relaxation of mesenteric arteries in response to AEA in female rats [97]; however, other experiments were performed on males.

The vasodilatory effects of (endo)cannabinoids (mainly their potency) depend on the hypertension model and vessel type (Table 2). Thus, the responses of resistance mesenteric arteries to AEA, MethAEA, and CBD were diminished in SHR [93,95,96] but enhanced in DOCA-salt [93,101] and unchanged in hypertension induced by chronic administration of L-NAME [98]. The only exception was the increase in potency but the decrease in the efficacy of the vasodilatory action of AEA in the mesenteric arteries of females [97]. In contrast, AEA showed stronger efficacy in the thoracic aorta of SHR [96] and rats with renal hypertension [100]. The vasodilatory effect of THC was enhanced in mesenteric arteries isolated from rats with hypertension induced by chronic L-NAME administration. Interestingly, small constriction and relaxation in the aorta in response to THC were noted in normotensive rats and rats with L-NAME-induced hypertension, respectively [99].

The most important mechanisms underlying the relaxant properties of (endo)cannabinoids are (1) stimulation of classical CBRs (CB_1_ and/or CB_2_), (2) stimulation of TRPV1 receptors, (3) activation of calcium channels, and (4) inhibition of calcium entry, along with (5) endothelium-dependent mechanisms (such as stimulation of hypothetical CB_X_ receptors) [59]. As shown in Table 2, a similar mechanistic approach can apply to hypertension. The most significant components of vascular response in this pathological condition are CBRs and endothelium. Interestingly, CB_1_Rs mostly participate only in the hypertensive response [95,101]. Similar effects of AEA and MethAEA suggest that AEA does not act via its metabolites in mesenteric arteries (Table 2).

**Table 2:** Direct acute effects of (endo)cannabinoids on arteries isolated from rats (if not otherwise specified) with systemic or pulmonary hypertension.

Compound	Model	Artery	E_max_ (%) (in Parentheses Concentrations in µM for Which E_max_ Was Obtained)		pEC_50_		Suggested Mechanism of Action in Hypertension	Ref.
			N	H	N	H		
AEA	WKY vs. SHR	perfused mesenteric bed	~100 ^1^ (10)	~100 ^1^ (10)	7.1	6.3 *	↓ NO-dependent relaxation; TRPV1-dependent	[96]
		G3 mesenteric	98 (3)	70 * (10)	6.5	6.8 *	sex-dependent (stronger in female); TRPV1- and endothelium-dependent	[97]
		thoracic aorta	13 (30)	48 * (30)	8.1	7.9	endothelium-dependent; CB_1_R- and TRPV1-independent	[96]
	L-NAME-induced	perfused mesenteric bed	100 (10)	107 (10)	6.5	7.1 *	-	[98]
			~90 ^1^ (10)	~90 ^1^ (10)	6.3	6.4	↑ sensory nerve-mediated activity	[96]
		G3 mesenteric	~70 ^1,2^ (30)	~70 ^1,2^ (30)	5.7	5.6	-	[98]
		thoracic aorta	25 (30)	33 (30)	6.7	6.6	CB_1_R-, TRPV1-, NO- and PG-independent	[96]
	2K1C	thoracic aorta	4 (30)	44 * (30)	-	5.2	CB_1_R-, CB_2_R-, NO- and endothelium-dependent	[100]
	hypoxia ^3^	isolated perfused lung	-	↑ pulmonary arterial tone (10)	-	-	FAAH-dependent metabolites;sex-dependent (stronger in females)	[102]
		large pulmonary	-	no effect (10)	-	no effect	-	
MethAEA	DOCA-salt	G3 mesenteric	84 (30)	85 (30)	4.9	5.6 *	TRPV1-dependent in N and H;CB_1_R-dependent in H only	[101]
		aorta	84 (30)	41 * (30)	6.1	n.d.	-	
	SHR	G3 mesenteric	97 (30)	98 (30)	6.1	5.6 *	CB_1_R-dependent in H only	[95]
	hypoxia ^3^	isolated perfused lung	-	no effect (10)	-	-	-	[102]
CBD	DOCA-salt	G3 mesenteric	92 (30)	91 (30)	5.5	5.9 *	CB_1_R-, CB_2_R- and endothelium-dependent	[93]
	SHR		93 (30)	82 (30)	6.0	5.6 *	CB_1_R-dependent; CB_2_R- and endothelium-independent	
	Hypertension ^4^	pulmonary	94 (30)	93 (30)	4.9	4.1 *	endothelium, PG- and TRPV1-dependent;CB_1_R-, CB_2_R-independent	
THC	L-NAME-induced	G3 mesenteric	~60 ^1^ (100)	~70 ^1^ (100)	5.6	6.1 *	CB_1_R-independent; ↑ sensory nerve-mediated activity and PG-dependent	[99]
		G0 mesenteric	16 (100)	38 * (100)	-	-	-	
		aorta	5—constriction (100)	4—relaxation (100)	-	-	-	

^1^ No precise data given, calculated from the figures in the publication. ^2^ Maximal effect was not determined. ^3^ Mouse model. ^4^ Human studies. * Significant difference at a level of at least *p* < 0.05 compared to normotension. n.d., not determined because of the too-low value of E_max_. ↑ increase; ↓ decrease; 2K1C—Goldblatt two-kidney, one-clip model; AEA—anandamide; CB_1_R—cannabinoid receptor type 1; CB_2_R—cannabinoid receptor type 2; CBD—cannabidiol; DOCA—deoxycorticosterone acetate; E_max_—maximal effect; FAAH—fatty acid amide hydrolase; G0—superior mesenteric artery (conduit); G3—third-order branches mesenteric artery (resistance); H—hypertension; L-NAME—L-N^G^-nitro arginine methyl ester; MethAEA—methanandamide; N—normotension; NO—nitric oxide; pEC_50_—the negative logarithm of the half maximal effective concentration; PG—prostanoids; Ref.—references; SHR—spontaneously hypertensive rat; THC—Δ^9-^tetrahydrocannabinol; TRPV1—transient receptor potential vanilloid 1; WKY—Wistar-Kyoto rat.

In addition to AEA, other eCBs and endocannabinoid-like compounds possess vasodilatory potencies, such as 2-AG, 2-AGE, ARA-S, NADA, NAGly, OEA, PEA, oleamide, and virodhamine [48,58]. However, they were not examined under hypertensive conditions. Sometimes they do not act directly but through their anti-inflammatory and vasodilatory ω-3 eCB epoxide regioisomer metabolites [103]. In addition, endocannabinoid-like compounds (e.g., OEA and PEA) [104] can also intensify the action of eCBs by competing with them for metabolizing enzymes, thus reducing their degradation (the so-called entourage effect) [38]. Interestingly, 2-AG induced contraction of rat aorta via vasoconstrictor metabolites [105]. The vascular activity of other eCBs and endocannabinoid-like compounds has not yet been examined.

## 8. Acute In Vivo Cardiovascular Effects of (Endo)cannabinoids

We previously reviewed the cardiovascular effects of (endo)cannabinoids in normotension [69] and systemic hypertension [46]. Briefly, the effects of eCBs on BP and heart rate (HR) are complex and vary depending on whether the animal is anesthetized or not [69]. In rats anesthetized with urethane, intravenous (*i.v.*) injection of AEA and its stable analog MethAEA resulted in a three-phase cardiovascular response. Phase I is characterized by rapid and marked bradycardia and a transient drop in BP (the so-called Bezold–Jarisch reflex), resulting from the activation of TRPV1 receptors located on cardiac afferents of the vagus fibers. It is not determined after acute *i.v.* administration of THC, CBD, or synthetic cannabinoids that do not activate TRPV1 receptors. Phase II (also observed after injection of MethAEA and THC) consists of a short-term pressure response (lasting approx. 30–60 s) associated with increased contractility of the heart and blood flow through the kidney and mesenteric bed. It results mainly from stimulation of the brain’s CB_1_Rs, glutamatergic NMDA, thromboxane A_2_ (TP), and β_2_-adrenergic receptors [69]. In phase III (also observed after injection of MethAEA, THC, and synthetic cannabinoids), there is a prolonged (up to 10 min) significant drop in BP, accompanied by decreased renal and mesenteric flow, a significant reduction in myocardial contractility, and a slight decrease in HR and vascular resistance. Phase III is suggested to result from [69]: (1) stimulation of presynaptic CB_1_Rs located at the ends of sympathetic fibers innervating blood vessels and the heart, inhibiting the release of norepinephrine; (2) stimulation of hypothetical CB_X_ endothelial vasodilating receptors; and (3) the CB_1_R-mediated negative inotropic effect of (endo)cannabinoids in the heart.

In conscious animals, the predominant effect of AEA, THC, and synthetic cannabinoid administration is the pressure response combined with the narrowing of the renal blood vessels and the mesentery. This mainly results from central activity [69]. Interestingly, an increase in arterial pressure, plasma noradrenaline concentration, and renal sympathetic tone has been observed after intracerebroventricular (*i.c.v.*) administration of synthetic cannabinoids or AEA in both anesthetized and conscious animals [69]. Similarly, stimulation of CB_1_Rs in the paraventricular nucleus of the hypothalamus (PVN) causes a pressor response in both anesthetized and conscious rats, clearly suggesting that central mechanisms are responsible for the increased BP induced by cannabinoids [71].

Unlike AEA, 2-AG caused only a monophasic response in the circulatory system of rats and pentobarbital- and/or urethane-anesthetized mice with hypotension and tachycardia, lasting about 10–18 min. However, the pressure drop observed does not depend on 2-AG itself, but on the arachidonic acid metabolites formed from 2-AG [69].

The endogenous endocannabinoid tone is not involved in regulating the cardiovascular system under physiological conditions since none of the CBR antagonists, inhibitors of eCBs metabolism, or genetic deletions of components of the endocannabinoid system modify cardiovascular parameters [69]. The situation is different under pathophysiological conditions [46]. For example, (1) acute *i.v.* injection of AEA and MethAEA induced stronger hypotension in anesthetized SHR as well as different models of secondary hypertension than in respective normotensive controls; and (2) two CB_1_R antagonists, rimonabant and AM251, further increased and two FAAH inhibitors, URB597 and AM3506, decreased the elevated BP and cardiac contractility in hypertensive animals and did not affect any hemodynamic parameters in normotensive controls.

Such promising results demonstrate the strong vasodilatory effects of (endo)cannabinoids in isolated resistance arteries (see Section 7) and the involvement of the endocannabinoid tone in cardiovascular system regulation in hypertension, and the more evident hypotensive response to these compounds in hypertension (see above) suggests potential beneficial therapeutic effects. Experiments with the chronic administration of (endo)cannabinoids allowed for verification of the above theory.

## 9. Cardiovascular Effects of Chronic (Endo)cannabinoid Administration in Hypertension

Table 3 shows the results from all publications regarding the influence of chronic administration of (endo)cannabinoids or compounds modifying the endocannabinoid tone on BP and HR in experimental models of hypertension and a few cases in human trials. Particular compounds were studied in both hypertensive and normotensive control groups. Importantly, the compounds did not significantly affect BP in normotensive individuals. The amplitude of changes in BP (both decreases and increases) depended on their basal values. The lack of changes in normotension can be explained by too low basal pressure. However, in experiments performed on isolated vessels (see Table 2 and Section 7), (endo)cannabinoids elicited full or almost full vasorelaxation of pre-constricted resistance arteries isolated from normotensive and hypertensive donors. Interestingly, cannabinoids affected HR in hypertension in only two cases [87,106], which indicates that different mechanisms are involved in the regulation of BP and HR. It should be remembered that the main effect of marihuana in humans is tachycardia, in contrast to the bradycardia noticed in animals after acute (endo)cannabinoid injection [76,78].

The first group of cannabinoids studied in hypertension was exogenously administrated eCBs or compounds inhibiting their metabolism. As shown in Table 3, only one studied endocannabinoid-like compound, PEA, confirmed the working hypothesis that a compound exerting strong vasodilatory activity [104] could also possess hypotensive potential after chronic application. Indeed, after 5 weeks of subcutaneous (*s.c.*) PEA administration in SHR rats [107,108], a strong hypotensive effect was noticed. The lack of such action before then (weeks 1–4) might have resulted not only from the vasodilatation but also from the protection against kidney injury (for details, see Section 10.4).

In contrast to distinct and prolonged hypotension observed after acute injection with the main eCB, AEA, or the inhibitor of its degradation, URB597, in hypertensive rats (see Section 8), such a promising effect was not noted after chronic administration (see Table 3). Thus, AEA tended to increase BP in Dahl salt-sensitive rats (with a high-salt diet) [109], while it decreased BP in SHR [110,111]. This discrepancy in the effects probably does not result from small differences in doses or procedure duration (3 vs. 5 mg/kg and 2 vs. 4 weeks, respectively) but from the form, route, and frequency of administration. Golosova et al. [109] experimented with *i.v.* AEA administration in its unmodified form once daily, whereas Martín Giménez et al. [110,111] used a nanoformulated compound and gave it intraperitoneally (*i.p.*) once weekly. Unaltered compounds with 100% bioavailability and no first-pass effect acted more strongly and aggressively, but for a shorter time because of their rapid metabolism. The nanoformulated version was released slowly, and the action was more delayed. Kidney injury has been suggested as the direct cause of the hypertensive effect of *i.v.* AEA (see Section 10.4), which might be induced by repeated administration of toxic concentrations of the compound. It is possible that a cardiotoxic effect of AEA described previously in vitro [112] could also occur in this model.

**Table 3:** Cardiovascular effects of chronic administration of (endo)cannabinoids in different models of systemic hypertension in male rats (unless noted otherwise).

Compound, Dose, and Protocol	Model	BP and HR Effects	Influence on Changes Induced by Hypertension		References
			Cardiac Effects/Expression in Heart (If Not Stated Otherwise)	Vascular Effects	
PEA 30 mg/kg, *s.c.*, once daily, 5 weeks	SHR	- ↓SBP (only in the 5th week of the treatment; by ~50–60 mmHg) - ↔HR	n.d.	vasodilatory effects in mesenteric or carotid arteries: - ↑EDHF-mediated relaxation to Ach; - ↑synthesis/release of vasodilatory EETs, NO, and PGI_2_ and/or ↓EETs degradation; - ↓RAAS activity (↓ACE and AT_1_R signaling pathway); anti-inflammatory effects: ↓NF-κB signaling pathway	[107,108]
AEA 3 mg/kg, *i.v.*, once daily, 14 days	Dahl salt-sensitive + high salt (8%) diet	- consistent trend to ↑MBP at the 2nd week of the treatment (by ~20 mmHg)	n.d.	n.d.	[109]
nf-AEA 5 mg/kg, *i.p.*, once weekly, 4 weeks	SHR	- ↓SBP after 4 weeks (by 35 mmHg) ^1^	anti-hypertrophic effects: ↓ventricular mass and LV hypertrophy indexes	n.d.	[110,111]
URB597 1 mg/kg, *i.p.*, twice daily, 14 days	DOCA-salt	- ↓SBP (after 2 weeks by ~30–60 mmHg) - ↔ HR	anti-hypertrophic effects: - ↓cardiac (only in younger) and LV hypertrophy - ↓medium and large coronary artery thickness in LV cardiac functional effects: - ↓diastolic stiffness - tendency to ↑cardiostimulatory effects of isoprenaline: contractility, cardiac work and inotropism - normalization of (-) inotropic effect of CB_1_R agonism anti-oxidant effects: ↓ROS, 4-HNE, CO gr., XO, NADPH oxidase activity and ↑GSH, GSH/GSSG, vit. C, ↑Nrf2, p21, ↓Keap1 pro-oxidant effects: ↓GSH-Px, GSSG-R, Cu-Zn-SOD, Trx-R activity and ↑MDA, 8-OHdG, ↓Trx, vit. A, ERK1/2, HO-1, MAPK pro-inflammatory effects: ↑TNFα endocannabinoid effects: - ↑FAAH in LV, ↓FAAH, MAGL activity - tendency to ↓CB_1_R and CB_2_R in LV but ↑CB_1_R and CB_2_R in whole heart - ↑TRPV1, GPR55, PPARα, ↓PPARγ - ↑NADA and 2-AG other effects: - ↑heart availability of energy substrates - ↑intramyocardial glycogen storage - ↓apoptosis (↓ Bax, caspase 3, 9)	vasodilatory effects: ↓response to phenylephrine in sMAs anti-hypertrophic effects: ↓medial thoracic aorta hypertrophy endocannabinoid effects: ↓FAAH in sMAs other effects: ↑K_Ca_3.1 sMAs	[101,113,114,115,116,117,118,119,120]
URB597 1 mg/kg, *i.p.*, twice daily, 14 days	SHR	- ↔SBP or slight ↓SBP (by ~20 mmHg after 2 weeks) and HR	hypertrophic effects: ↑heart hypertrophy but ↓LV hypertrophy cardiac functional effects: - ↑(+) chronotropic effect of isoprenaline - normalization of (+) inotropic effect of isoprenaline in atria anti-oxidant effects: ↓XO, ↑CAT, Trx-R activity, ↑GSH, GSH/GSSG, vit. E, C, Trx, ↓Keap1, Bach1, ↑ERK1/2, MAPK pro-oxidant effects: ↓GSH-Px activity and ↑MDA, 4-HNE, 8-isoprostanes, 8-OHdG, CO gr., ↓Nrf2, Keap1, HO-1 anti-inflammatory effects: ↓TNFα endocannabinoid effects: ↑CB_1_R, CB_2_R, GPR55, PPARγ and ↓TRPV1, PPARα - translocation of CB_1_R immunoreactivity to the intercalated discs in LV - tendency to ↑FAAH in LV - ↓FAAH and MAGL activity - ↑AEA, NADA, and 2-AG other effects: - ↑cardioprotective LV sphingolipid (S1P) - ↑palmitate uptake by LV cardiomyocytes - protection from DAG and CER accumulation in LV - improvement of insulin signaling in LV - ↓free AA - ↓apoptosis (↑Bcl-2, ↓Bax, caspase 3, 8, 9)	vasodilatory effects - ↓phenylephrine-mediated CB_1_R-independent vasoconstriction in sMAs - ↑potency of Ach-mediated endothelium-dependent vasorelaxation in sMAs and aorta - ↑potency of MethAEA-mediated CB_1_R-independent vasorelaxation vasoconstrictive effects: ↑vasoconstrictive potency of U46619 (thromboxane analog) in sMAs anti-hypertrophic effects: tendency to ↓sMAs wall hypertrophy endocannabinoid effects - ↑2-AG in aorta, ↑AEA in sMAs and aorta - ↓CB_1_R in aorta	[95,116,117,120,121,122]
JZL195 10 mg/kg, *i.p.*, once daily, 14 days	SHR	- tendency to ↓BP (by ~20 mmHg after 2 weeks) - ↔HR	- no changes in cardiac hypertrophy	n.d.	[123]
rimonabant 20 mg, oral, once daily, 12 months	hypertension ^2^	- ↓SBP by ~13 and 7 mmHg and DBP by ~6 and 2 mmHg in H. and N. patients, respectively	n.d.	n.d.	[124]
rimonabant 20 mg, oral, once daily, 12 months	hypertension ^2^	- ↓SBP by ~3 and 0.5 mmHg and DBP by ~2 and 0.5 mmHg in H. and N. patients, respectively	- reductions more evident in patients with higher cardiometabolic risk (e.g., dyslipidemia and type 2 diabetes) - the hypotensive effect seems to be mediated by weight loss		[125]
rimonabant 20 mg, oral, once daily, 24 months	hypertension ^2^	- ↓SBP by ~1.5 and 0.5 mmHg and DBP by ~2 and 0.5 mmHg in H. and N. patients, respectively	- changes not significantly different from placebo		[126]
rimonabant 10 mg/kg, oral, once daily, 3 weeks	(mRen2)27 higher RAAS activity	- ↓SBP (by ~25 mmHg within 24 h and remained lower through 3 weeks); ↔HR - better sympathetic and parasympathetic baroreflex sensitivity	n.d.	n.d.	[127]
LH-21 1 mg/kg, 3 mg/kg, *i.p.*, 3 weeks	KKAγ mice (BP was ↑ by about 10 mmHg only) ^3^	- normalization of SBP, DBP, MBP (only for 3 mg/kg) - ↔HR	n.d.	anti-inflammatory effects on aorta: - ↓ICAM-1, MCP-1, TNFα mRNA - ↓lipocalin-2	[128]
JWH133 1 mmol/l, 10 µL, *i.c.v.*, once daily, 4 weeks	SHR (conscious and anesthetized)	- ↓MBP and HR by ~35 mmHg and 70 beats/min respectively after 2 weeks of administration	n.d.	n.d.	[106]
O-1602 0.25 mg/kg, *i.a.*, once daily, 14 days	SHR ^3^	- ↓MBP by ~30 mmHg ^1^ - ↑HR by ~50 beats/min ^1^	n.d.	other effects: ↓RhoA/Rho-kinase signaling in aorta	[87]
CBD 10 mg/kg, *i.p.*, once daily, 14 days	DOCA-salt	- ↔HR, SBP, DBP, and MBP	anti-hypertrophic effects: ↓width of LV cardiomyocytes cardiac functional effects - ↓carbachol-induced vasoconstriction of coronary arteries - ↑(-) inotropic effect of CB_1_R agonism - ↑lusitropic effects: (+) isoprenaline and (-) carbachol anti-oxidant effects: ↓MDA, ↓GSSG, ↑GSH and small ↓4-HNE pro-oxidant effects: small ↓vit. A and E endocannabinoid effects: - ↓2-AG, OEA, DEA, DGLEA - ↓FAAH activity - small ↓CB_1_R, CB_2_R, and GPR18 other effects: - ↑FFA LA and ↓ FFA AA - ↓β_1_-adrenoceptor in LV	vasodilatory effects: - ↑Ach-induced endothelium-dependent vasorelaxation in aortas (NO-dependent) and sMAs - ↑eNOS in aortas and sMAs, ↑*NOS3* in sMAs, ↑*PGIS* in sMAs anti-hypertrophic effects: ↓aorta and sMAs hypertrophy endocannabinoid effects: - ↓CB_1_R in sMAs but ↑*Cnr1* in aortas - ↑*Cnr2* in aortas and sMAs - ↑AEA, 2-AG, PEA, and DEA; tendency to ↑OEA, HEA, POEA, LEA, and 2-LG; ↓EPEA, DHEA, and NAGly in aorta other effects: - ↓vWF in aortas and sMAs - ↑*KCNN4* in aortas and sMAs - ↑*KCNN3* in sMAs	[94,129,130]
CBD 10 mg/kg, *i.p.*, once daily, 14 days	SHR	- ↔HR, SBP, DBP, and MBP	anti-hypertrophic effects: ↓width of LV and RV myocytes and ↓RV hypertrophy cardiac functional effects - small ↓diastolic stiffness - ↓carbachol-induced vasoconstriction of coronary arteries - ↓(-) inotropic effect of CB_1_R agonism - ↑lusitropic effects: (+) isoprenaline and (-) carbachol anti-oxidant effects: ↓4-HHE and tendency to ↓4-HNE, ↑GSH, and ↓GSSG pro-oxidant effects: ↓vit. A and E endocannabinoid effects: - small ↓FAAH activity - ↓GPR55 and small ↓CB_1_R and GPR18 other effects: ↑FFA LA, FFA AA	vasodilatory effects: - ↑Ach-induced endothelium-dependent vasorelaxation in aortas and sMAs (COX dependent) - ↑eNOS in aortas and sMAs, ↑*NOS3* in aortas and sMAs, ↑*PGIS* in sMAs vasoconstrictive effects: ↓potency of SNP-induced vasorelaxation in sMAs anti-hypertrophic effects: ↓aorta and sMAs hypertrophy pro-inflammatory effects: ↑COX-1 in aorta endocannabinoid effects: - ↑CB_1_R in sMAs and tendency to ↑*Cnr1* in aortas and sMAs - ↑*Cnr2* in aortas and sMAs - ↑TRPV1 in aortas - ↓AEA and small ↓2-AG, PEA, HEA, DEA, EPEA, DHEA, LEA, 2-LG, and NAGly in aortas other effects: - ↓vWF in aortas and sMAs - ↑*KCNN4* in aortas and sMAs - ↑*KCNN3* in sMAs	[94,129,130]
CBD 200 mg/kg, oral, 4 weeks	OLETF rats with metabolic syndrome	- ↔BP ^1^	- loss of visceral adiposity was not associated with reduced BP		[131]
Δ^8^-THC 3 mg/kg, *i.p.*, once daily, 14 days	ARH unilaterally adrenalectomized +1% NaCl ^3^	- ↓BP (by ~13 and 15 mmHg at the end of the 1st and 2nd week)	n.d.	n.d.	[132]
Δ^9^-THC 3 mg/kg, *i.p.*, once daily, 7 or 14 days		- ↓BP (by ~18 and 13 mmHg at the end of the 1st and 2nd week); - tolerance to the acute hypotensive effect of the compound (in a shorter protocol)	n.d.	n.d.	
Δ^9^-THC 1 mg/kg 2 mg/kg, *s.c.*, once daily, 3–5 weeks	metacorticoid and renal hypertension	- ↔BP and HR	n.d.	n.d.	[133]
Δ^9^-THC 5–25 mg/kg (increasing dosing), oral, once daily, 5 or 10 days	SHR	- transient ↓BP after increasing the dose (tolerance developed) - ↓SBP after highest dose chronic treatment (with no tolerance effect)	n.d.	n.d.	[134,135]

The Table summarizes all significant effects described in particular publications. Non-significant results are not mentioned. ^1^ BP and HR were determined at endpoint only. ^2^ In humans, SBP was less than 165 mmHg and DBP less than 105 mmHg. ^3^ Female animals. ↑ increase; ↓ decrease; ↔ no effect; *i.a.*—intraarterial; *i.c.v.*—intracerebroventricular; *i.g.*—intragastrical; *i.p.*—intraperitoneal; *i.v.*—intravenous; *s.c.*—subcutaneous; 2-AG—arachidonoylglycerol; 2-LG—2-linoleoylglycerol; 4-HHE—-hydroxyhexenal; 4-HNE—4-hydroxynonenal; 8-OHdG—8-hydroxy-2′-deoxyguanosine; AA—arachidonic acid; ACE—angiotensin-converting enzyme; Ach—acetylcholine; AEA—anandamide; ARH—adrenal regeneration hypertension; AT1R—angiotensin II type 1 receptor; Bach1—BTB and CNC homology 1 transcription factor; Bax—pro-apoptotic bcl-2-like protein 4; Bcl-2—B-cell lymphoma 2; BP—blood pressure; CAT—catalase; CB_1_R—cannabinoid receptor type 1; CB_2_R—cannabinoid receptor type 2; CBD—cannabidiol; CER—ceramide; *Cnr1*—gene encoding CB_1_R protein; *Cnr2*—gene encoding CB_2_R protein; CNS—central nervous system; CO gr.—protein carbonyl groups; COX—cyclooxygenase; Cu-Zn-SOD—cytosolic superoxide dismutase; DAG—diacylglycerol; DBP—diastolic blood pressure; DEA—docosatetraenoyl ethanolamide; DGLEA—dihomo-γ-linolenoyl ethanolamide; DHEA—docosahexaenoyl ethanolamide; DOCA—deoxycorticosterone acetate; EDHF—endothelium-derived hyperpolarizing factor; EETs—epoxyeicosatrienoic acids; eNOS—endothelial nitric oxide synthase; EPEA—eicosapentaenoyl ethanolamide; ERK—extracellular signal-regulated kinases; FAAH—fatty acid amide hydrolase; FFA—free fatty acids; GPR—G protein-coupled receptor; GSH—glutathione; GSH-Px—glutathione peroxidase; GSSG—glutathione disulfide; GSSG-R—glutathione reductase; H—hypertensive; HEA—homo-γ-linolenyl ethanolamide; HO-1—heme oxygenase 1; HR—heart rate; ICAM-1—intercellular adhesion molecule 1; *KCNN3*—gene encoding K_Ca_2.3 protein; *KCNN4*—gene encoding K_Ca_3.1 protein; Keap1—kelch-like ECH-associated protein 1; KKAγ mice, spontaneously diabetic; LA—linoleic acid; LEA—linolenoyl ethanolamide; LV—left ventricle; MAGL—monoacylglycerol lipase; MAPK—mitogen-activated protein kinase; MBP—mean blood pressure; MCP-1—monocyte chemoattractant protein-1; MDA—malondialdehyde; MethAEA—methanandamide; N—normotensive; NADA—N-arachidonoyl dopamine; NADPH—nicotinamide adenine dinucleotide phosphate; NAGLy—N-arachidonoyl glycine; n.d.—not determined; NF-κB—nuclear factor kappa-light-chain-enhancer of activated B cells; nf-AEA—nanoformulated anandamde; NO—nitric oxide; *NOS3*—gene encoding eNOS; Nrf2—nuclear factor erythroid 2-related factor 2; OEA—oleoyl ethanolamide; OLETF—Otsuka Long-Evans Tokushima Fatty type 2 diabetic rats; p21—cyclin-dependent kinase inhibitor 1; PEA—palmitoyl ethanolamide; PGI_2_—prostacyclin; *PGIS*—gene encoding prostacyclin synthase; POEA—palmitoleoyl ethanolamide; PPAR—peroxisome proliferator-activated receptors; RAAS—renin-angiotensin-aldosterone system; ROS—reactive oxygen species; RV—right ventricle; S1P—sphingosine-1-phosphate; SBP—systolic blood pressure; SHR—spontaneously hypertensive rat; sMAs—small mesenteric arteries (resistance); SNP—sodium nitroprusside; THC—tetrahydrocannabinol; TNFα—tumor necrosis factor α; TRPV1—transient receptor potential vanilloid 1; Trx—thioredoxin; Trx-R—thioredoxin reductase; vit.—vitamin; vWF—von Willebrand factor; XO—xanthine oxidase.

Chronic administration of the FAAH inhibitor URB597, which mainly degrades AEA, modified BP in a model-dependent manner. In secondary DOCA-salt hypertension, it decreased BP after 2 weeks of treatment [113,114,115,116,119], whereas in SHR (primary hypertension), there was no change [116,117] or only a slight decrease [121]. This was probably due to the more dynamic development of hypertension in DOCA-salt vs. SHR (4 weeks vs. 8–10 weeks to obtain similar BP values). An alternative explanation has to do with model-dependent vasodilatory effects of (endo)cannabinoids in isolated vessels. As shown in Table 2, both MethAEA and CBD caused mesenteric vasodilatation, which was more potent in DOCA-salt hypertensive than in control animals, whereas in SHR, these effects were weaker than in normotension. Another inhibitor, JZL195, which inhibits both FAAH and MAGL and stops AEA and 2-AG degradation, only showed a tendency to lower BP in SHR [123]. This suggests that 2-AG does not intensify the hypotensive effect of AEA observed after URB597 administration.

Activation of CB_1_Rs might increase BP via central effects or decrease BP via direct vasodilatation, reduce noradrenaline release from sympathetic nerve endings innervating resistance vessels, or decrease cardiac contractility [45,69]. The direct synthetic CB_1_R antagonist rimonabant, acting nonspecifically on both the peripheral and central level, was also investigated as a potential antihypertensive agent. It was examined in a big clinical trial, Rimonabant in Obesity (RIO), mostly including obese, diabetic, or dyslipidemia patients. The results obtained for the extracted hypertensive group showed that one-year [124,125] or two-year [126] treatment with rimonabant resulted in only small decreases compared to normotension. However, it should be noted that only patients with BP below 165 mmHg were enrolled in the trial. Moreover, the hypotensive effect could be caused by weight loss.

In an animal model of (mRen2), 27 rats (a monogenetic model of Ang II-dependent hypertension in which the mouse renin *Ren2* gene is transfected into the Sprague–Dawley rat genome), a higher dose of rimonabant (10 mg/kg vs. 20 mg in a clinical trial) caused a significant pressure drop [127]. Except for the difference in dose, in the animal experiment, there was also higher basal pressure. Importantly, the hypotensive effect appeared as early as 24 h after CB_1_R antagonist administration and remained lower for 3 weeks of examination. Interestingly, acute *i.v.* rimonabant injection increased BP in SHR [136] but decreased it in (mRen2)27 rats [127], again proving that the potential hypotensive effects of (endo)cannabinoids are model-dependent. Isolated peripheral blockade of CB_1_Rs by LH-21 normalized slightly increased BP in spontaneous diabetic KKAγ mice [128]. Thus, the beneficial effect of antagonizing CB_1_Rs also has a peripheral component. However, the fact that antagonists of CB_1_Rs were effective in hypertension contradicts the use of compounds that stimulate these receptors in this indication, including the aforementioned eCBs and/or compounds that increase their concentration. What is more, antagonists of CBRs caused an effect that was more explicit and intense. Unfortunately, the compounds stimulating CBR and CB_1_R antagonists were examined in different models of hypertension.

Other single targets studied in hypertensive animals (SHR in both cases) were CB_2_R and GPR55 receptors. 28-day-lasting *i.c.v.* administration of CB_2_R agonist JWH133 resulted in a distinct fall in BP [106]. A similar reduction in BP occurred when O-1602, a GPR55 receptor (and to a lesser extent GRP18) agonist was used intra-arterially (*i.a.*) for 2 weeks [87]. Interestingly, the influence of chronic administration of compounds affecting the endocannabinoid system on HR was noted only in these two cases (JWH133 decreased it, and O-1602 increased it in hypertensive animals).

As shown in Table 3, the potential hypotensive influence of chronic administration of two phytocannabinoids was also examined in experimental hypertension. The first one, CBD at a dose of 10 mg/kg administered over 2 weeks, failed to diminish BP in both DOCA-salt and SHR [129]. Even a much higher dose of CBD (200 mg/kg) did not improve BP-related effects in OLETF rats with mild obesity, the clinical onset of diabetes mellitus, and metabolic syndrome [131]. Better effects were found with Δ^8^- and Δ^9^-THC; however, there is variability among performed studies. Low *s.c.* dose (1 mg/kg) of Δ^9^-THC did not alter BP in metacorticoid or renal hypertension [133]. A higher dose (3 mg/kg) given *i.p.* was effective in ARH for both Δ^8^- and Δ^9^-THC, although a longer scheme (14 days) did not lead to tolerance induction [132]. The highest doses of Δ^9^-THC (5–25 mg/kg), administered orally, resulted in a stable decrease in BP after the highest dose [135] and transient lowering of pressure after increasing the lower dose in SHR, after which tolerance was induced [134].

We could not determine whether the effects induced by chronic (endo)cannabinoid administration are gender-dependent since most of the experiments were performed on male animals, and none of the compounds have been studied under comparable conditions in both sexes (see Table 2 and Table 3).

The choice of route of administration in the described studies should not be surprising. The authors mostly used *i.p.* and *s.c.* injections, and in only a few cases (mostly in clinical trials) oral administration. These are the easiest to perform and give the full dose of the administered compound, although they are unlikely to be translated into clinical trials and further into clinical practice. So, if a compound shows promising effects, it should be tested using a more approachable route of administration: oral or inhalation. The latter is especially interesting since it is the most common route for recreational cannabis use and also for many cannabinoid-based drugs [137,138]. To date, no studies on chronic hypertension with inhaled (endo)cannabinoids have been performed. However, we would like to point out that THC increases HR in humans independent of its route of administration (including inhalation, oral, or even *i.v.*) [78], so the effects of the examined (endo)cannabinoids may also stay the same regardless of their formulation.

Unfortunately, so far, there is no publication regarding the influence of chronic cannabis use, either recreationally or therapeutically, in patients with hypertension. We can only suppose that, similar to the results obtained using experimental hypertension models, their final effect on BP would depend on whether they stimulate one or more targets. Moreover, it should be kept in mind that (1) there are species differences (e.g., acute administration of THC causes tachycardia in humans and bradycardia in experimental animals) [78], and (2) marijuana and synthetic cannabimimetics can induce acute myocardial infarction (MI) in healthy young people [78]. For example, a recent analysis of the UK Biobank dataset demonstrated that cannabis use was a statistically significant positive predictor for MI [139].

## 10. Potential Mechanisms of Cardiovascular Effects of Chronic (Endo)cannabinoid Administration in Hypertension

**Figure 2:**
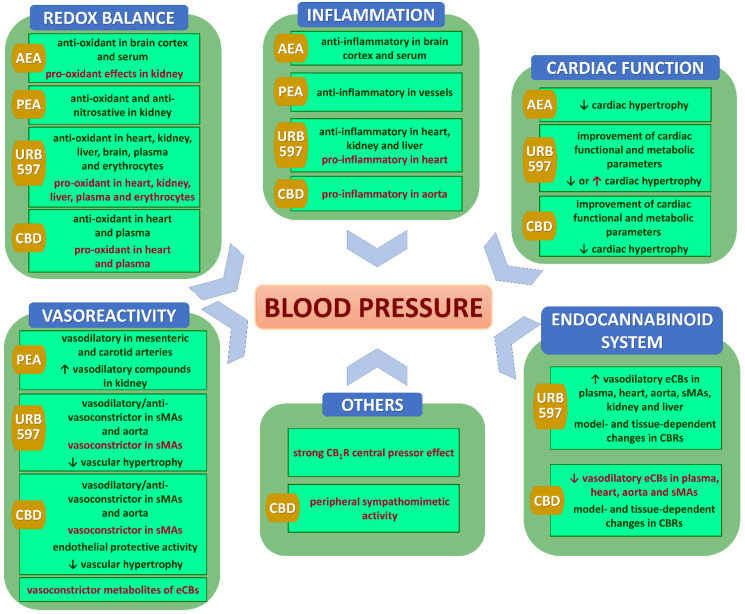
Summarized effects of multitarget (endo)cannabinoids on blood pressure. For clarity, the effects, listed in detail in Table 3 and Table 4, are partly simplified and are based on results of all parameters connected with particular mechanisms, which sometimes were opposite. ↑—increase; ↓—decrease; AEA—anandamide; CBRs—cannabinoid receptors; CB_1_R—cannabinoid type 1 receptor; CBD—cannabidiol; eCBs—endocannabinoids; PEA—palmitoyl ethanolamide; sMAs—small mesenteric arteries.

As shown in Table 3 and Table 4 and Figure 2 and listed below, several potential mechanisms of antihypertensive effects were investigated in the examination of cardiovascular effects of chronic (endo)cannabinoid administration in various hypertension models. The tables summarize only significant effects described in particular publications; non-significant results are not mentioned. In the description below and Figure 2, we include only the most important mechanisms listed in the modified Dr. Page’s Mosaic Theory of hypertension [8] (see Section 5) and the most intensively studied after chronic (endo)cannabinoid administration.

### 10.1. Vasodilatation

The strong vasodilating effects of (endo)cannabinoids in isolated vessels, depending on the hypertension model, have been described (Section 7 and Table 2). Notably, chronic administration of (endo)cannabinoids enhanced some vasorelaxant action (mostly in resistance arteries) via the following mechanisms: (1) improvement of the vasodilator effect elicited by Ach and/or MethAEA observed after chronic treatment with PEA [108] and URB597 [95] in SHR, and CBD in both DOCA-salt and SHR [94]; (2) reduction in vasoconstrictor response to phenylephrine in DOCA-salt [101] and SHR [95] under chronic FAAH inhibition; (3) enhancement of vasodilating compound synthesis (such as epoxyeicosatrienoic acids (EET), NO, and PGI_2_) or decrease in RAAS activity in vessels [94,108]; and (4) decrease in aortic hypertrophy and/or sMAs in SHR and DOCA-salt hypertensive animals treated with URB597 or CBD [94,95,101].

On the contrary, in some cases, pro-constrictive effects were observed, such as increased vasoconstriction induced by thromboxane A_2_ analog or decreased response of the vasorelaxant sodium nitroprusside (SNP) after chronic URB597 [95] and CBD [94] administration, respectively, observed in sMAs of SHR. These effects may at least partially counteract the compounds’ beneficial effects on hypertension.

### 10.2. Cardiac Functional Antihypertensive Effects

Several beneficial changes in cardiac functional parameters were noted after chronic cannabinoid treatment: (1) decreased diastolic stiffness after URB597 in DOCA-salt [116] and CBD in SHR [130], (2) improved cardiostimulatory isoprenaline influence (positive inotropic and lusitropic effects under chronic URB597 [116] and CBD [130] treatment, respectively), (3) normalized cardiac negative inotropic effect of CB_1_R agonist CP55940 (only in DOCA-salt rats) after both URB597 and CBD, and (4) diminished carbachol-induced vasoconstriction of coronary arteries after chronic CBD administration in DOCA-salt and SHR [130]. In addition to the functional improvements, cannabinoids were potent in diminishing left ventricle (LV) overgrowth, the most prominent hypertrophic effect of systemic hypertension. The effectiveness was demonstrated by nf-AEA [110], URB597 [116,121], and CBD [130]. A similar anti-hypertrophic effect was observed in the kidneys of DOCA-salt animals treated with URB597 [119]. Since many place the kidney at the center of the pathobiology of systemic hypertension [8], this could be the reason for the better reaction to URB597 treatment in DOCA-salt.

### 10.3. Changes in Endocannabinoid System Components

The hypotensive effect or lack of an effect may also be induced by changes in eCBs released in different tissues. eCBs with proven vasodilating properties were characterized before (see Section 7).

As shown in Table 3 and Table 4, changes in eCBs distribution have been studied after chronic treatment with URB597 and CBD only. The effectiveness of treatment was confirmed by decreased FAAH activity in various tissues, as well as for CBD, which inhibits this enzyme [49]. URB597 also diminished MAGL activity in the heart, mesenteric artery, kidney, and liver of DOCA-salt and/or SHR (Table 3 and Table 4). Using these two hypertension models allowed us to demonstrate that changes in the levels of eCBs and their receptors are mainly tissue- and model-dependent. URB597 acted more uniformly than CBD. It mostly increased the levels of potentially vasorelaxant eCBs in plasma (AEA and NADA in DOCA-salt and SHR; 2-AG in SHR), heart (AEA in SHR; NADA and 2-AG in DOCA-salt and SHR), aorta (AEA and 2-AG in SHR), sMAs (AEA in SHR), kidneys (AEA, 2-AG, NADA in both models), or liver (AEA and NADA in SHR). In contrast, CBD mainly decreased eCB levels in the heart (2-AG, OEA) and plasma (AEA) in DOCA-salt and plasma (small PEA, OEA) in SHR. In the aorta, it also reduced NAGly levels in DOCA-salt and AEA in SHR, and tended to diminish levels of 2-AG, PEA, and NAGly in SHR. On the other hand, it increased concentrations of AEA, 2-AG, PEA, and DEA in the aortas of DOCA-salt animals. In the case of CBD, changes in the levels of other compounds with so far unknown vasodilatory potentials, such as DEA, DGLEA, LEA, EPEA, DHEA, HEA, and 2-LG, in various tissues of hypertensive animals have been determined.

Besides activating or blocking various receptors, cannabinoids may self-regulate their action by altering the expression of classical and non-classical CBRs in the tissues. As shown in Figure 1, activation of those receptors should result in beneficial effects, so an increase in expression is considered positive and a decrease negative. A different situation occurs where CB_1_Rs are concerned because they may evoke both protective and damaging processes. After URB597 treatment of DOCA-salt rats, an increase in CB_2_Rs and TRPV1 (heart, kidney), GPR55, and PPARα (heart) and a decrease in CB_1_Rs (kidney, tendency in LV) were observed. On the other hand, an increase of CB_1_Rs in the heart and liver and a decrease of PPARα in the liver and PPARγ receptors in the heart occurred [113,116,117,142]. Quite different changes happened in the SHR model. The expression of CB_2_R (heart, kidney), GPR55 (heart, brain), TRPV1 (liver), and PPARγ (heart) receptors increased, and CB_1_R decreased in the aorta but increased in the heart and kidney, whereas the expression of CB_2_R (liver, brain), TRPV1 (heart), and PPARα (heart) receptors decreased [117,140,142,144]. Chronic administration of CBD also elicited model-dependent changes in receptor expression. CB_1_R expression decreased in the heart and sMAs, but increased in the aorta; CB_2_R expression decreased in the heart but increased in sMAs and aorta; and GPR18 decreased in the heart in DOCA-salt animals. In SHR, CB_1_R expression decreased in the heart but increased in sMAs and aorta, CB_2_R expression increased in sMAs and aorta, GPR18 decreased in the heart, and TRPV1 increased in the aorta [94,129]. To summarize, as listed above, the effects of URB597 and CBD on the expression of various receptors are tissue- and model-dependent. However, it seems that, in general, beneficial effects dominate over negative ones.

### 10.4. Anti- and Pro-Oxidative Effects

Known anti- and pro-oxidative effects of activation/blockade of CBRs (see Figure 1), as well as direct inhibitory action of CBD affecting oxidative and nitrosative stress [145], implicate them as possible mechanisms involved in the regulation of BP [8,62].

Indeed, as shown in Table 3 and Table 4, depending on the administration protocol and hypertension model, AEA caused anti-oxidant effects in CNS and serum (less frequent administration of the nanoformulated form in SHR) [111] and pro-oxidants in the kidney (frequent *i.v.* dosing in Dahl salt-sensitive animals) [109]. In these two cases, post-treatment oxidative status corresponded to changes in BP, i.e., decrease and increase, respectively. In contrast to AEA, a pronounced anti-oxidant effect of PEA in the kidney is postulated as one of the main mechanisms responsible for the pressure drop following chronic administration of this compound [107].

Chronic URB597 administration caused ambiguous oxidative effects in hypertension (Table 3 and Table 4). In both DOCA-salt and SHR, it resulted in almost the same intense pro- and anti-oxidative impact on heart tissue [117,120], which was also confirmed in rat plasma [117], erythrocytes [141], kidney [142], and liver [113,144]. The only clear anti-oxidant effect was observed in the SHR brain [140], which did not lead to a fall in BP (small or no antihypertensive effect; Table 3).

CBD, well known for its anti-oxidant (mostly direct) properties [145], showed not unequivocal but rather positive modifications in the redox balance of hypertensive rats [129]. However, given the lack of an antihypertensive effect, the outcome was either too weak or counteracted by other opposing effects.

### 10.5. Anti-Inflammatory Effects

Inflammation is also inextricably linked to oxidative stress in hypertension [8]. As shown in Table 3 and Table 4, chronic (endo)cannabinoid administration exerts mainly anti-inflammatory effects. Unfortunately, inflammatory parameters have been examined relatively rarely. Importantly, anti-inflammatory consequences in hypertension support previously described anti-oxidant effects of PEA (mesenteric bed) [108] and nf-AEA (CNS and serum) [111]. URB597 treatment mostly showed effects against inflammation in cardiac tissue [120], kidney [142], and liver [113,144]. The use of CB_1_R antagonists [128] or CB_2_R agonists [106] also resulted in decreased inflammation (in the aorta and CNS, respectively), which could explain the hypotensive effect of the above compounds. Importantly, it was demonstrated recently that marijuana smoking elevated plasma markers of inflammation associated with atherosclerosis and that THC-induced inflammation, oxidative stress, and endothelial dysfunction in mice were responsive to the CB_1_R antagonist genistein [139].

### 10.6. Other Pro-Hypertensive Effects

The mechanisms described above do not always fully explain the presence or absence of the hypotensive effect of (endo)cannabinoids. The question arises as to what other factors, sometimes only literature-based, could reduce the potential hypotensive effects of chronically administered compounds.

One factor could be central CB_1_Rs, activation of which is responsible for the pressor effect. As mentioned in Section 8, *i.v.* injection of (endo)cannabinoids decreased BP in anesthetized animals but increased it in conscious animals. Microinjection of (endo)cannabinoids into the PVN enhanced BP in anesthetized and conscious rats, and chronic administration of the CB_1_R antagonist rimonabant decreased BP (Table 2). These three effects suggest that the central mechanisms responsible for the increased BP induced by cannabinoids may be superior to those involved in hypotension (at least in some models of hypertension).

Another aspect that should be noted is that acute *i.v.* injection of CBD strongly increased SBP and HR but decreased DBP in pithed rats (a model that allows examination of peripheral effects only since the animals’ CNS is destroyed). Enhancement of both of these cardiovascular parameters was evoked by the peripheral sympathomimetic activity of CBD; the lower DBP was probably related to the direct vasodilatory properties of CBD. Two opposite effects are probably responsible for CBD at 10 mg/kg not affecting cardiovascular parameters within 1 h after *i.p.* administration in conscious rats [146].

It should also be kept in mind that the well-known vasodilatory action of eCBs may sometimes be diminished by their vasoconstrictor metabolites, e.g., OEA [58] and AEA, which is even suggested as a PH enhancer (for details, see Section 12) [102]. Similarly, 2-AG can act differently on the vessels (through vasodilation or vasoconstriction) [48,58] and can also have opposite effects on the heart (protective or damaging) [147,148].

## 11. Why Multitarget Vasodilatory (Endo)cannabinoids Are Not Effective as Antihypertensive Compounds

To summarize, Table 3 and Table 4 show the effects of chronic administration of monotarget (rimonabant, LH-21, JWH133, and O-1602) and multitarget (PEA, AEA, URB597, JZL195, CBD, and THC) (endo)cannabinoids on systemic hypertension. We included O-1602 in the monotarget group since it has a higher affinity for GPR55 than GPR18 receptors [149], and other multitarget compounds act by at least three different targets (e.g., CBD, 65 targets) [150]. Except for CBR antagonists and inhibitors of enzymes responsible for eCB degradation, all compounds possess proven vasodilatory properties, in many cases also in hypertension (Table 2 and Section 7), and were shown to decrease BP more strongly in anesthetized hypertensive rats than normotensive rats after acute *i.v.* administration (Section 8). It should be emphasized that all monotarget (endo)cannabinoids are synthetic ones. Among multitarget compounds, synthetic, phyto-, and endocannabinoids can be found. (Endo)cannabinoid origin (synthetic, plant-derived, or endogenous) is not, therefore, an indicator of its potential beneficial action in hypertension.

Chronic administration of all monotarget substances caused a significant fall in BP. However, experiments were conducted on only one model of hypertension in each study. What is more, very specific routes of administration (*i.a.* for O-1602, *i.c.v.* for JWH133), rather impossible to translate into human therapy, were used. In addition, a clinical trial of rimonabant in obese patients was conducted, in which an extracted group of individuals with hypertension showed decreased BP with the compound. Still, it is not certain whether the effect was due to weight loss. Besides, rimonabant was withdrawn from the market due to serious side effects [39].

The results considering chronic administration of multitarget (endo)cannabinoids are more complicated. AEA increased or decreased BP, URB597 caused a small, model-dependent drop in BP or had no hypotensive effect, and CBD failed to modify BP regardless of the model used. Only PEA clearly decreased BP in SHR. However, this effect was noticed only in the fifth week of administration. Interestingly, similar to PEA, a delayed hypotensive response was observed with the other compounds (for details, see Table 3), which rather excludes the direct influence of vasodilatation as the main reason for their influence on BP.

Figure 2, which outlines various influences of multitarget compounds on BP in hypertension, is an attempt to answer the main question of why multitarget vasodilatory (endo)cannabinoids are not effective as antihypertensive compounds. They can lead to a fall in BP as a result of not only direct vascular relaxation but also the release of various vasorelaxant compounds, the enhancement of such action elicited by other endogenous substances (e.g., Ach), the release of vasodilatory eCBs or decreased vasoconstrictor activity (e.g., phenylephrine), and reduced cardiac and vessel hypertrophy and anti-oxidant and anti-inflammatory capacity in various tissues.

However, chronic AEA, URB597, or CBD administration can also stimulate effects leading to increased BP. First of all, it should be kept in mind that (endo)cannabinoids produce complex cardiovascular effects and that central CB_1_Rs are also responsible for stimulating the distinct pressor response (for details, see Section 6). AEA is a potent CBR agonist. CBD, well known as a negative allosteric modulator of CB_1_Rs, can also stimulate this receptor. Recently, central CB_1_Rs have been demonstrated as a target in CBD action in anxiety, in a manner sensitive to rimonabant and absent in CB_1_^-/-^ mice [150]. Moreover, eCBs can also cause vasoconstriction via their metabolites. Additionally, the model- and tissue-dependent influence on sensitivity to cannabinoid receptors might also determine the direction of changes in BP since stimulation of CB_1_Rs enhances oxidative and inflammatory states (see Figure 1). Thus, after chronic URB597 and CBD treatment, some pro-vasoconstriction changes were observed. Importantly, the anti-oxidant activity of these two compounds was accompanied by an almost equally intense pro-oxidative effect. URB597 also showed a slight pro-inflammatory effect, partly interfering with its overall anti-inflammatory properties. The same is true for CBD, a known anti-inflammatory compound, which showed minor inflammatory activity. In the case of CBD, two additional observations should be taken into consideration: (1) it reduced the level of vasodilatory eCBs; (2) it possesses peripheral sympathomimetic activity (for details, see Section 10.6). Finally, the model- and tissue-dependent influence on sensitivity to cannabinoid receptors might also determine the direction of changes in BP since stimulation of CB_1_Rs enhances oxidative and inflammatory states (see Figure 1).

In summary, monotarget compounds seem more beneficial as potential antihypertensive drugs than multitarget compounds. In this context, synthetic monotarget cannabinoids should have an advantage over endocannabinoids, which do not have such precise sites of action. However, monotarget compounds were examined in one hypertension model only, specific routes of administration (*i.a.* or *i.c.v*.) were used, and the CB_1_R antagonist rimonabant, which had been examined in long-term clinical studies, was withdrawn from the market because of its undesirable side effects. Thus, further experiments with monotarget cannabinoids are needed to determine the best compounds. The first single experiments with agonists of CB_2_ and GPR55 receptors and with a peripheral CB_1_R antagonist are encouraging. The bad experience with rimonabant excludes the recommendation of other first-generation CB_1_R antagonists (that cross the blood–brain barrier), although central CB_1_Rs responsible for the pressor effect seem to strongly counteract the peripheral vasodilatory effect anyway. In light of this, the third generation of CB_1_R antagonists, i.e., peripherally restricted dual-target CB_1_R antagonists (e.g., hybrid CB_1_R antagonist and inducible NOS inhibitor) [39], remains to be examined.

## 12. In Vivo Effects of Chronic (Endo)cannabinoids in PH

As shown in Table 2 of the review by Krzyżewska et al. [48], all main components of the endocannabinoid system (AEA, 2-AG, CB_1_Rs, CB_2_Rs, TRPV1, GPR18, GPR55 receptor, and FAAH) are present in the pulmonary circulation or lung tissue. Importantly, eCBs AEA, 2-AG, virodhamine, the endogenous agonists of GPR55 (l-alpha-lysophosphatidylinositol (LPI)) and GPR18 (NAGly) receptors caused full or almost full relaxation of pre-constricted human pulmonary arteries [48].

However, in contrast to its potent vasodilatory activity, AEA is postulated to mediate hypoxia-induced pulmonary vasoconstriction [102] based on the following facts: (1) hypoxia stimulated AEA synthesis in pulmonary arterial smooth muscle cells in vitro; (2) AEA (but not 2-AG) increased pulmonary arterial tone in isolated perfused mouse lungs via its vasoconstrictor metabolites (Table 2); (3) genetic FAAH deletion or chronic administration of FAAH inhibitor URB597 prevented the onset of PH (Table 5). The beneficial influence of FAAH inhibition could result from the inhibition of vasoconstrictor metabolite synthesis or the enhancement of AEA and its protective action, neither of which was determined under in vivo conditions. Notably, the vasoconstriction effect of AEA on isolated perfused mouse lungs was more pronounced in female animals (Table 2), which is in line with the statistic that PH is more common in women.

**Figure 3:**
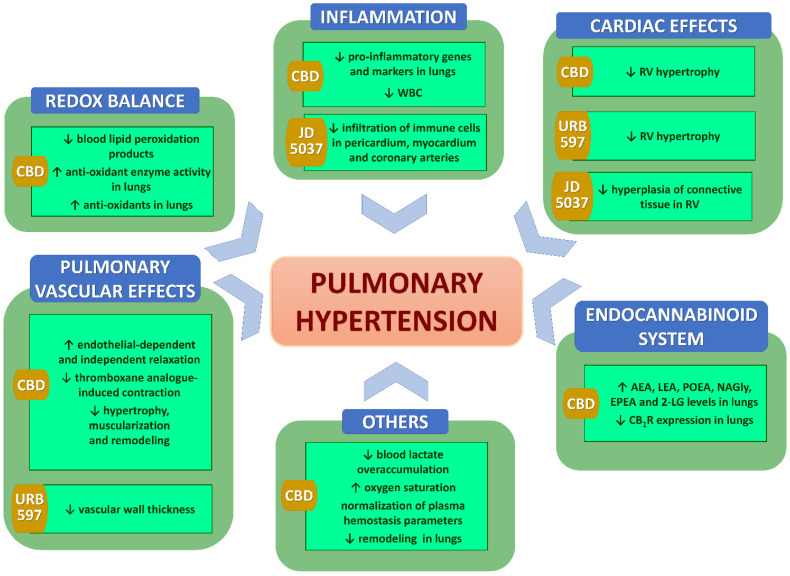
Summarized effects of (endo)cannabinoids on pulmonary hypertension. For clarity, the effects, listed in detail in Table 5, are partly simplified. ↑—increase; ↓—decrease; 2-LG—2-linoleoylglycerol; AEA—anandamide; CB_1_R—cannabinoid type 1 receptor; CBD—cannabidiol; EPEA—eicosapentaenoyl ethanolamide; LEA—linolenoyl ethanolamide; NAGly—N-arachidonoyl glycine; PA—pulmonary artery; POEA—palmitoleoyl ethanolamide; RV—right ventricle; WBC—white blood cells.

As shown in Table 5 and Figure 3, except for the paper by Wenzel et al. mentioned above, the chronic effects of (endo)cannabinoids on PH have only been examined in the last two years. Importantly, all those studies revealed the positive effects of the administered drugs. First of all, there was a significant decrease in right ventricular systolic pressure (RVSP), the main parameter determining the severity of the disease. This is very interesting since the authors used different, sometimes contrary, targets. As mentioned above, FAAH inhibition prevented PH development [102]. On the other hand, the peripheral CB_1_R antagonist JD5037 alone tended to lower RVSP only in the MCT-induced model of rat PH. Still, it potentiated the effect of metformin in a combined therapy protocol [151]. Thus, the roles of AEA and CB_1_Rs remain to be examined in detail.

The richest data available are for phytocannabinoid CBD. It has been used in two models of PH, the Sugen/hypoxia mouse model [152] and the rat MCT model [152,153]. Two protocols were applied: 14-day treatment or 21-day preventive in the former, and 21-day preventive in the latter. In both, CBD caused a strong drop in RVSP. Comparable effects of CBD in CB_2_R knockout mice and their wild-type littermates confirmed the lack of involvement of those receptors in its protective action [152]. In addition, CB_1_Rs were found to not participate in the anti-PH activity of CBD [48].

All experiments investigating chronic cannabinoids in PH showed anti-hypertrophic effects of the compounds (Table 5). The most common were decreased Fulton’s index, which indicates hypertrophy of RV induced by increased afterload and reduced vascular hypertrophy. CBD also altered PA reactivity (intensified response to relaxants and diminished response to constrictors) [153]. The mechanism of action was examined in more detail for CBD only. Protection against changes induced by PH might be based on anti-inflammatory or anti-oxidant action in blood and lungs [152,153]. Additionally, CBD increased pulmonary levels of some eCBs with vasodilatory effects on PA [153]. Furthermore, studies on PH reported an influence on systemic BP in both normotensive and PH groups.

As with systemic hypertension, studies mostly used routes of administration that are convenient (*i.p.*, *i.g.*), but this would not fully meet the expectations of possible future clinical practice. An interesting solution in the case of PH would be administration by inhalation [137,138]. This could produce not only a systemic response but also (or maybe only) a local effect in the lung tissue, which is known to be the center of the disease. Importantly, treatment delivered by inhalation is already being used in therapy for PAH (treprostinil), with a good isolated effect on pulmonary vasculature [2]. On the other hand, results obtained in a randomized controlled trial demonstrated that single-dose inhalation of vaporized cannabis did not modify the airway function in patients with advanced chronic obstructive pulmonary disease (COPD) [154].

**Table 5:** Effects of chronic administration of cannabinoids on various tissues of male animals (unless noted otherwise) in different models of pulmonary hypertension.

Compound, Dose, and Protocol	Model	Effects			Ref.
**CARDIOVASCULAR**					
		**BP and HR Effects**	**Influence on Changes Induced by Hypertension**		
			**Cardiac Effects/Expression in Heart** **(If Not Stated Otherwise)**	**Vascular Effects**	
FAAH^−/−^ in comparison to WT	hypoxia (mice) ^1^	- no ↑RVSP	hypertrophic effects: no ↑Fulton index	hypertrophic effects: no ↑vascular wall thickness	[102]
URB597 5 mg/kg, *i.p.*, once daily, 3 days or 3 weeks		- ↓RVSP (in longer procedure) (by ~5 mmHg)	anti-hypertrophic effects: ↓Fulton index (in longer procedure)	anti-hypertrophic effects: ↓vascular wall thickness (in longer procedure)	
JD5037 3 mg/kg, oral, once daily, 3 weeks	MCT (rat)	- intensification of the metformin-induced ↓RVSP - ↔BP; ↔HR	anti-hypertrophic effects: ↓hyperplasia of connective tissue in myocardium anti-inflammatory effects: - ↓infiltration of immune cells in pericardium, myocardium, and coronary arteries other effects: - ↓vacuolization of tunica media of coronary arteries	-	[151]
CBD 10 mg/kg, 20 mg/kg, *i.g.*, once daily, 14 days (treatment) or 3 weeks (preventive)	SuHx/ SuHx *Cnr2*^-/-^ (mice)	- ↓RVSP (by ~10 mmHg)	anti-hypertrophic effects: ↓Fulton index	anti-hypertrophic effects: - ↓PA hypertrophy - ↓PA muscularization - ↓remodeling (PCNA^+^/nuclei)	[152]
CBD 10 mg/kg, *i.p.*, once daily, 3 weeks (preventive)	MCT (rat)	- ↓RVSP (by ~15 mmHg) - ↔BP; ↔HR	anti-hypertrophic effects: small ↓Fulton index	vasodilatory effects in PA: - ↑endothelial-dependent (Ach) and endothelial-independent (SNP) relaxation - ↓thromboxane analog-induced contraction anti-hypertrophic effects in PA: - ↓hypertrophy - ↓muscularization - ↓remodeling (PCNA^+^/nuclei)	[152,153]
**BLOOD**					
CBD 10 mg/kg, *i.g.*, once daily, 3 weeks (preventive)	SuHx (mice)	anti-oxidant effects: ↓blood MDA other effects: ↓blood lactate overaccumulation			[152]
CBD 10 mg/kg, *i.p.*, once daily, 3 weeks (preventive)	MCT (rat)	anti-inflammatory effects: ↓WBC other effects: - ↑oxygen saturation - normalization of plasma hemostasis parameters (↓PAI-1 and t-PA levels)			[153]
**LUNGS**					
CBD 10 mg/kg, *i.g.*, once daily, 3 weeks (preventive)	SuHx (mice)	anti-oxidant effects: ↑GSSG-R and GSH-Px activity anti-inflammatory effects: ↓*Il6* and *Tnfα* other effects: ↓lactate accumulation (↓*Pfkfb3*)			[152]
CBD 10 mg/kg, *i.p.*, once daily, 3 weeks (preventive)	MCT (rat)	anti-oxidant effects: ↑TAC, GSH, GSSG-R activity anti-inflammatory effects: ↓NFκB, TNFα, MCP-1, IL-1β, CD68 endocannabinoid effects: - ↑AEA, LEA, POEA, NAGLy, EPEA, and 2-LG; ↓CB_1_R other effects: ↓Gal-3			[153,155,156]

The Table summarizes all significant effects described in particular publications. Non-significant results are not mentioned. ^1^ Female animals. ↑ increase; ↓ decrease; ↔ no effect; *i.g.*—intragastrical; *i.p.*—intraperitoneal; 2-LG—2-linoleoylglycerol; Ach—acetylcholine; AEA—anandamide; BP—blood pressure; CB_1_ R—cannabinoid receptor type 1; CBD—cannabidiol; CD68—cluster of differentiation 68; *Cnr2^-/-^*—knockout of gene encoding CB_2_ R protein; EPEA—eicosapentaenoyl ethanolamide; FAAH—fatty acid amide hydrolase; Gal-3—galectin 3; GSH—glutathione; GSH-Px—glutathione peroxidase; GSSG-R—glutathione reductase; HR—heart rate; IL—interleukin; *Il6*—gene encoding IL-6 protein; LEA—linolenoyl ethanolamide; MCP-1—monocyte chemoattractant protein-1; MCT—monocrotaline; MDA—malondialdehyde; NAGLy—N-arachidonoyl glycine; NF-κB—nuclear factor kappa-light-chain-enhancer of activated B cells; PA—pulmonary artery; PAI-1—plasminogen activator inhibitor 1; PCNA—proliferating cell nuclear antigen; *Pfkfb3*—gene encoding 6-phosphofructo-2-kinase/fructose-2,6-biphosphatase 3 enzyme; POEA—palmitoleoyl ethanolamide; RVSP—right ventricular systolic pressure; SNP—sodium nitroprusside; SuHx—sugen/hypoxia model; t-PA—tissue plasminogen activator; TAC—total antioxidant capacity; TNFα—tumor necrosis factor α; *Tnfα*—gene encoding TNFα protein; WBC—white blood cells; WT—wild type.

In summary, CBD appears more effective against pulmonary than systemic hypertension (see Section 9). The question is how to explain it. In both types of hypertension, the authors used the same dose (10 mg/kg; a higher dose was not better in PH studies) and a similar route of administration (*i.p.*; intragastric (*i.g.*) only in experiments on mice). The potential beneficial effect of CBD on systemic hypertension was examined only with the use of a therapeutic (14-day) protocol, while for PH, both therapeutic (14-day) and preventive (21-day) protocols were used. The therapeutic scheme used might be the reason for the lack of the compound’s effectiveness in systemic hypertension since it is more difficult to reverse disease progression than to prevent its development. Interestingly, the effects of CBD in systemic hypertension were model-dependent, while a comparable influence of CBD in two PH models was observed. It should be kept in mind that the pulmonary and systemic vasculature have uniquely distinct roles and features; the pulmonary circulation is a low-resistance, high-capacity circuit with the advantage of local regulatory mechanisms, whereas systemic blood vessels are high-resistance, low-capacity conduits. In addition, the peripheral sympathomimetic effect of CBD determined in systemic hypertension (see Section 10.6) may not play an important role in PH since it was mainly observed as a cardiac component (increased HR) which was not observed in PH models.

## 13. Conclusions

Our review summarizing publications regarding chronic administration of (endo)cannabinoids in experimental models of hypertension demonstrates that the best outcomes in systemic hypertension were obtained using a few monotarget compounds. In contrast, chronic administration of multitarget (endo)cannabinoids failed to modify higher BP, and they are not recommended for the treatment of systemic hypertension since they induce responses leading to both decreased and increased BP (for details, see Figure 2).

The best results in PH were obtained with chronic administration of CBD (the only compound examined in detail), which was effective in two PH models and two treatment protocols (preventive and therapeutic). Since significant differences exist between the systemic and pulmonary vasculature and the pathophysiology of systemic and pulmonary hypertension, it seems reasonable to examine other (endo)cannabinoids (including multitarget) against PH.

Importantly, in chronic preclinical experiments on normo- and hypertension, (endo)cannabinoids were found to be rather safe compounds, with no serious adverse effects (except in the aggressive AEA *i.v.* administration protocol), so they can be used for other indications.

To summarize, other preclinical and clinical studies are still needed to determine the beneficial role of vasodilator (endo)cannabinoids in systemic (only monotarget) or pulmonary (both mono- and multitarget) hypertension.

## Figures and Tables

**Table 1:** Short list of characteristics of chosen models of systemic and pulmonary hypertension.

Type of Hypertension		Model	Main Characteristics
**Systemic**	**Primary**	SHR	- early age development (starting from 3–4 weeks) - ↑sympathetic activity - RAAS overactivation - ↑arterial wall stiffness - immune alterations
		Dahl salt-sensitive rat	- low-renin hypertension - kidney injury - ↓responses to vasorelaxants and ↑to vasoconstrictors
		TGR(mRen2)27	- suppression of RAAS with high prorenin levels
	**Secondary**	Ang-II	- RAAS-dependent hypertension - overactivity of the sympathetic nervous system - BP-independent kidney injury - vascular pressor/remodeling activity
		L-NAME	- NOS-deficient hypertension - systemic and renal vasoconstriction - renal interstitial fibrosis and glomerulosclerosis - immune alterations
		DOCA-salt	- low-renin hypertension - suppression of RAAS - severe renal and cardiac complications - remodeled aortic wall - ↑inflammatory signaling
		ARH	- potassium depletion - electrolyte disturbances - renal deficiency
		metacorticoid hypertension	- similar to DOCA-salt - more stable hypertension development
		renal hypertension (2K1C)	- RAAS overactivation
**Pulmonary**		MCT	- pulmonary vascular damage - remodeling and ↑vascular resistance - RV failure - intense perivascular inflammation - parenchymal alterations - no plexiform lesions
		hypoxia	- pulmonary vascular remodeling - RV hypertrophy - absence of RV failure - enhanced pulmonary vasoconstriction - no plexiform lesions
		sugen/hypoxia	- PH more stable than in hypoxia model - presence of RV failure - with plexiform lesions

For respective references, see Section 4. ↑ increase; ↓ decrease; 2K1C—two-kidney, one clip; Ang-II—angiotensin II; ARH—adrenal regeneration hypertension; DOCA—deoxycorticosterone acetate; L-NAME—L-N^G^-nitro arginine methyl ester; MCT—monocrotaline; NOS—nitric oxide synthase; PH—pulmonary hypertension; RAAS—renin-angiotensin-aldosterone system; RV—right ventricle; SHR—spontaneously hypertensive rat.

**Table 4:** Effects of chronic administration of (endo)cannabinoids in various tissues of different models of systemic hypertension in male rats (unless noted otherwise).

Compound, Dose, and Protocol	Model	Effects	References
**CENTRAL NERVOUS SYSTEM**			
nf-AEA 5 mg/kg, *i.p*., once weekly, 4 weeks	SHR	anti-inflammatory/-oxidant effects: ↓WT-1, AT_1_R, iNOS, and ↑Hsp70 in brain cortex other effects: ↓apoptosis (TUNEL and caspase-3) in brain cortex	[111]
URB597 1 mg/kg, *i.p.*, twice daily, 14 days	SHR	anti-oxidant effects in brain: - ↑Cu-Zn-SOD, GSH-Px, GSSG-R activity, ↓MDA, ↑vit. E - ↑Nrf2 and HO-1 and ↓Bach1 endocannabinoid effects in brain: - ↓FAAH activity and ↑AEA - ↓CB_2_R and ↑GPR55 other effects: ↓phospholipid but ↑free AA, DHA, and LA in brain	[140]
JWH133 1 mmol/l, 10 µL, *i.c.v.*, once daily, 4 weeks	SHR	anti-inflammatory effects: ↓IL-1β, IL-6, and TNFα in RVLM	[106]
**BLOOD**			
nf-AEA 5 mg/kg, *i.p.*, once weekly, 4 weeks	SHR	anti-inflammatory effects: ↓IL-1, IL-6, TNFα, uCRP, and Hsp70 in serum anti-oxidant effects: ↓NADPH oxidase serum activity and ↑nitrites (an indirect measure of NO) in serum	[111]
URB597 1 mg/kg, *i.p.*, twice daily, 14 days	DOCA-salt	anti-oxidant effects: ↑GSH, ↓MDA in plasma, and ↓MDA in erythrocytes pro-oxidant effects: ↓ plasma GSH-Px activity endocannabinoid effects: - ↑AEA and NADA but ↓2-AG in plasma - ↓CB_1_R, CB_2_R, TRPV1, GPR55 in lymphocytes other effects: - ↑plasma insulin and ↑insulin sensitivity (HOMA-IR, QUICKI, and FGIR) - ↑anti-aggregation effect (↑sialic acid in erythrocytes, sialic acid in plasma and ↑negative charge of the erythrocyte membrane) - normalization of electrochemical properties of erythrocyte; ↓erythrocyte size - ↓phospholipid AA and ↑free AA, DHA, LA in plasma - ↑phospholipids in erythrocytes membrane (PC, PS, and PE)	[115,117,118,141]
URB597 1 mg/kg, *i.p.*, twice daily, 14 days	SHR	anti-oxidant effects: ↑GSSG-R plasma activity and ↓MDA in erythrocytes pro-oxidant effects: ↑plasma ROS, MDA, and ↓GSH in erythrocytes endocannabinoid effects: - ↑AEA, NADA, and 2-AG in plasma - ↑TRPV1 and ↓CB_2_R in lymphocytes other effects: - ↓plasma insulin and ↓ insulin sensitivity (HOMA-IR) - ↑anti-aggregation effect (↑sialic acid in erythrocytes, ↓sialic acid in plasma and ↑negative charge of the erythrocyte membrane) - normalization of electrochemical properties of erythrocyte, ↓erythrocyte size - ↓phospholipid DHA in plasma, ↑phospholipids in erythrocytes membrane (PC, PS, PE, and PI)	[117,122,141]
rimonabant 10 mg/kg, oral, once daily, 4 weeks	(mRen2)27	other effects: ↓serum leptin and insulin	[127]
CBD 10 mg/kg¸ *i.p.*, once daily, 14 days	DOCA-salt	anti-oxidant effects: ↑vit. E, GSH, ↓MDA, and tendency to ↓GSSG and 4-HHE in plasma pro-oxidant effects: small ↓plasma GSH-Px and GSSG-R activity endocannabinoid effects: ↓AEA and LEA in plasma	[129]
CBD 10 mg/kg, *i.p.*, once daily, 14 days	SHR	anti-oxidant effects: ↓CO gr., tendency to ↑GSH, ↓GSSG, and 4-HNE in plasma pro-oxidant effects: small ↓plasma GSH-Px activity endocannabinoid effects: ↓SEA, HEA, DGLEA and tendency to ↓PEA, OEA, LEA in plasma other effects: ↓free AA in plasma	[129]
**KIDNEY**			
AEA 3 mg/kg, *i.v.*, once daily, 14 days	Dahl salt-sensitive + high salt (8%) diet	pro-oxidant effects: ↓Nrf2 in renal cortex other effects: - ↑Smad3 in renal cortex and ↑interstitial fibrosis and glomeruli damage score - ↑Ca^2+^ excretion on day 7	[109]
PEA 30 mg/kg, *s.c.*, once daily, 5 weeks	SHR	vasodilatory effects: - ↑vasodilatory metabolites (HETEs and EETs) synthesis and/or ↓their degradation - ↓RAAS activity (↓AT_1_R, ↑AT_2_R signaling pathway) anti-oxidant and anti-nitrosative effects: - ↓ROS, MDA and ↑Cu-Zn-SOD and p47phox - ↓iNOS and protein nitrotyrosylation - small ↓urinary MDA and nitrite other effects: ↑urinary output - ↓severity of glomerulosclerosis and tubulointerstitial fibrosis	[107]
URB597 1 mg/kg, *i.p.*, twice daily, 14 days	DOCA-salt	anti-hypertrophic effects: ↓renal hypertrophy (only in younger rats) anti-oxidant effects: ↓ROS, XO, NADPH oxidase, Trp and ↑GSH-Px, GSSG-R activity, ↑GSH, vit. A, p-cJun, ↓Keap1 pro-oxidant effects: ↓Cu-Zn-SOD, CAT activity and ↑4-HNE, MDA, 8-OHdG and ↓p21 and HO-1 anti-inflammatory effects: ↓TNFα and ↓COX-1 and COX-2 activity endocannabinoid effects: ↓FAAH and MAGL activity - ↑AEA, 2-AG, and NADA; ↓CB_1_R, ↑ CB_2_R, and TRPV1 other effects: ↑free AA, DHA, and phospholipid AA - intensification of changes induced by hypertension	[119,142,143]
URB597 1 mg/kg, *i.p.*, twice daily, 14 days	SHR	anti-oxidant effects: ↓ROS, XO, CO gr.; ↑Cu-Zn-SOD activity, GSH, vit. E, A, HO-1 pro-oxidant effects: ↓GSH-Px activity, ↑4-HNE, MDA, NPs, 8-OHdG, Keap1, Bach1, ↓p21 anti-inflammatory effects: ↓COX-1, COX-2 activity pro-inflammatory effects: ↑cPLA2 activity endocannabinoid effects: ↓FAAH and MAGL activity - ↑AEA, 2-AG, and NADA; ↑CB_2_R and CB_1_R other effects: ↑free AA and DHA - prevention of changes in electrical properties of the cell membrane, sialic acid, and protein content	[142,143]
rimonabant 10 mg/kg, oral, once daily, 4 weeks	(mRen2)27	other effects: ↑urine osmolality (at day 21)	[127]
**LIVER**			
URB597 1 mg/kg, *i.p.*, twice daily, 14 days	DOCA-salt	anti-oxidant effects: ↓XO, NADPH oxidase, ↑Cu-Zn-SOD, GSH-T activity, ↑GSH, GSSG, vit. A, ↓Trp, Keap1, Bach1, ↑p-cJun pro-oxidant effects: ↓GSSG-R activity, vit. E, p21, ERK1/2, HO-1, ↑4-HNE, MDA, 4-ONE, 8-OHdG, dityrosine anti-inflammatory effects: ↓NFκB, TNFα endocannabinoid effects: ↓FAAH and MAGL activity - ↓2-AG, ↑ CB_1_R, and ↓ PPARα other effects: ↓phospholipid DHA and LA - ↓ apoptosis (↓caspase 3, 9 but ↑ caspase 8)	[113]
URB597 1 mg/kg, *i.p.*, twice daily, 14 days	SHR	anti-oxidant effects: ↓XO, NADPH oxidase, ↑CAT, GSH-Px activity, p21, p-ERK1/2, HO-1, ↓ CO gr. pro-oxidant effects: ↓GSSG-R activity, ↑MDA, 8-OHdG, Keap1, Bach1, ↓ p-cJun, Trx anti-inflammatory effects: ↓NFκB, TNFα, and ↑COX-2 endocannabinoid effects: ↓FAAH activity - ↑AEA, NADA, ↓ CB_2_R, and ↑TRPV1 other effects: ↓phospholipid AA, free AA, and ↑ free DHA, LA	[144]
Δ^8^-THC, Δ^9^-THC 3 mg/kg, *i.p.*, once daily, 14 days	ARH unilaterally adrenalectomized +1% NaCl ^1^	hypertrophic effects: ↑liver hypertrophy/weight	[132]

The Table summarizes all significant effects described in particular publications. Non-significant results are not mentioned. ^1^ Female animals. ↑ increase; ↓ decrease; *i.c.v.*—intracerebroventricular; *i.p.*—intraperitoneal; *i.v.*—intravenous; *s.c.*—subcutaneous; 2-AG—arachidonoylglycerol; 4-HHE—4-hydroxyhexenal; 4-HNE—4-hydroxynonenal; 4-ONE—4-oxononenal; 8-OHdG—8-hydroxy-2′-deoxyguanosine; AA—arachidonic acid; AEA—anandamide; ARH—adrenal regeneration hypertension; AT_1_R—angiotensin II type 1 receptor; AT_2_R —angiotensin II type 2 receptor; Bach1—transcription regulator protein BACH1; CAT—catalase; CB_1_R—cannabinoid receptor type 1; CB_2_R—cannabinoid receptor type 2; CBD—cannabidiol; CO gr.—protein carbonyl groups; COX—cyclooxygenase; cPLA2—cytosolic phospholipase A2; Cu-Zn-SOD—cytosolic superoxide dismutase; DGLEA—dihomo-γ-linolenoyl ethanolamide; DHA—docosahexaenoic acid; DOCA—deoxycorticosterone acetate; EETs—epoxyeicosatrienoic acids; ERK—extracellular signal-regulated kinases; FAAH—fatty acid amide hydrolase; FGIR—fasting glucose/insulin ratio; GPR—G protein-coupled receptor; GSH—glutathione; GSH-Px—glutathione peroxidase; GSH-T—glutathione transferase; GSSG—glutathione disulfide; GSSG-R—glutathione reductase; HEA—homo-γ-linolenyl ethanolamide; HETEs—hydroxyeicosatetraenoic acids; HO-1—heme oxygenase 1; HOMA-IR—homeostasis model assessment of insulin resistance; Hsp70—70 kilodalton heat shock protein; IL—interleukin; iNOS—inducible nitric oxide synthase; Keap1—kelch-like ECH-associated protein 1; LA—linoleic acid; LEA—linolenoyl ethanolamide; MAGL—monoacylglycerol lipase; MDA—malondialdehyde; NADA—N-arachidonoyl dopamine; NADPH—nicotinamide adenine dinucleotide phosphate; nf-AEA—nanoformulated anandamde; NF-κB—nuclear factor kappa-light-chain-enhancer of activated B cells; NO—nitric oxide; NPs—neuroprostanes; Nrf2—nuclear factor erythroid 2-related factor 2; OEA—oleoyl ethanolamide; p-cJun—phosphorylated transcription factor Jun; p21—cyclin-dependent kinase inhibitor 1; p47phox—neutrophil cytosolic factor 1; PC—phosphatidylcholine; PE—phosphatidylethanolamine; PEA—palmitoyl ethanolamide; PI—phosphatidylinositol; PPAR—peroxisome proliferator-activated receptors; PS—phosphatidylserine; QUICKI—quantitative insulin sensitivity check index; RAAS—renin-angiotensin-aldosterone system; ROS—reactive oxygen species; RVLM—rostral ventrolateral medulla; SEA—stearoyl ethanolamide; SHR—spontaneously hypertensive rat; Smad3—mothers against decapentaplegic homolog 3; THC—tetrahydrocannabinol; TNFα—tumor necrosis factor α; Trp—tryptophan; TRPV1—transient receptor potential vanilloid 1; Trx—thioredoxin; TUNEL—terminal deoxynucleotidyl transferase dUTP nick end labeling; uCRP—ultrasensitive C-reactive protein; vit.—vitamin; WT-1—Wilms’ tumor-1 transcription factor; XO—xanthine oxidase.

## Data Availability

Not applicable.
